# Substitution reactions in the acenaphthene analog of quino[7,8-*h*]quinoline and an unusual synthesis of the corresponding acenaphthylenes by *tele*-elimination

**DOI:** 10.3762/bjoc.20.24

**Published:** 2024-02-08

**Authors:** Ekaterina V Kolupaeva, Narek A Dzhangiryan, Alexander F Pozharskii, Oleg P Demidov, Valery A Ozeryanskii

**Affiliations:** 1 Department of Organic Chemistry, Southern Federal University, Zorge str. 7, 344090 Rostov-on-Don, Russian Federation; 2 Department of Chemistry and Pharmacy, North Caucasus Federal University, Pushkin str. 1a, 355017 Stavropol, Russian Federation

**Keywords:** dipyrido[3,2-*e*:2′,3′-*h*]acenaphthene (acenaphthylene), hydrogen bonding, π-stacking, substitution reactions, *tele*-elimination

## Abstract

The possibility of functionalization of dipyrido[3,2-*e*:2′,3′-*h*]acenaphthene containing a quino[7,8-*h*]quinoline fragment and being a highly basic diazine analog of 1,8-bis(dimethylamino)naphthalene (“proton sponge”) has been studied for the first time. In addition to the pronounced tendency of the title compound to form associates with an intramolecular hydrogen bond of the NHN type (new examples with the participation of pyridine rings, including self-associates are shown) and its inertness to amination reactions of the pyridine rings, the naphthalene core at positions 5(8) and the CH_2_CH_2_ bridge (dehydrogenation) undergo chemical modifications under mild conditions, giving the corresponding acenaphthylenes. The latter can also be obtained in an unusual way by *tele*-elimination from 5,8-dibromodipyridoacenaphthene by reaction with neutral or anionic bases.

## Introduction

Quinoline derivatives, classical nitrogen-containing heterocycles, are widely distributed in nature in various forms and used in medicine, food industry, catalysts, dyes, functional materials, oil refining, and electronics [1–2]. Quinoline and its derivatives have antibiotic, antimalarial, antitumor, anti-inflammatory, antihypertensive, and antiretroviral properties [3–4]. Therefore, at present, there is a need for compounds containing a quinoline fragment in various fields of research.

**Scheme 1:**
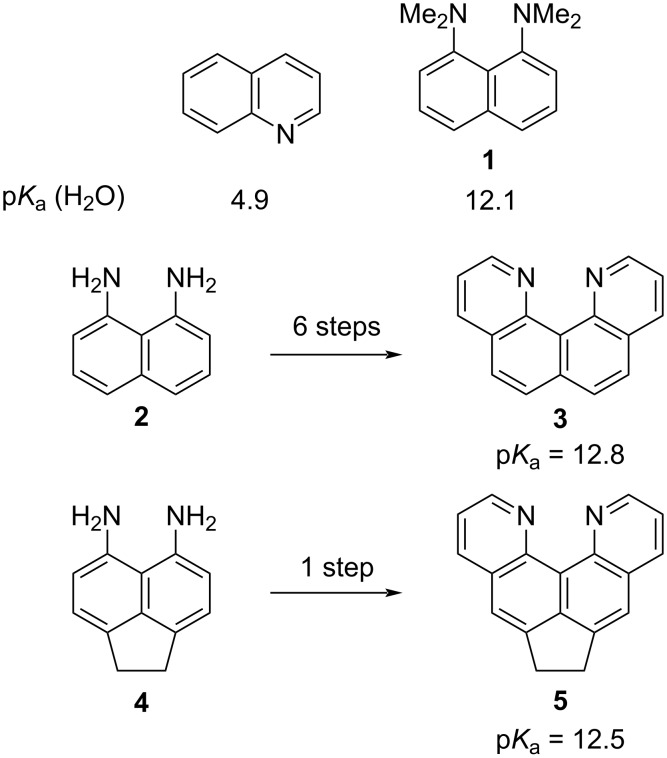
Comparison of basicity (in water scale) and synthetic availability of quinoline-type azaarenes and "proton sponge" **1**.

At the same time, quinoline bases are a popular platform for the molecular design of polycyclic systems with receptor properties; they easily form proton complexes with high stability and selectivity [5–6]. This, in turn, attracts the attention of researchers involved in the study of various types of hydrogen bonds and the problem of superbasicity [7–8]. Indeed, the basicity of quinoline and simple azaarenes is rather low. At the same time, the correct structural organization of azaarenes, where unshared electron pairs are forced to strongly repel each other in space, can lead to a sharp increase in basicity [8]. Thus, quino[7,8-*h*]quinoline (**3**), first obtained in the Staab group [9], already exceeds in basicity not only quinoline itself, but also 1,8-bis(dimethylamino)naphthalene (**1**, “proton sponge”) (Scheme 1; p*K*_a_ values of the corresponding monoprotonated forms are given). Meanwhile, the very synthesis of polycondensed quinoline bases with a certain arrangement of nitrogen atoms is often a challenge for a synthetic scientist [9–10]. This greatly limits the possible use of such polynuclear azaarenes in organic synthesis and the study of their properties.

For example, quinoquinoline **3** is synthesized in six steps from diamine **2** with a total yield not exceeding 10% [9]. This azaarene still remains a poorly available compound despite the rich chemistry of its functional derivatives, the interesting tautomeric, proton acceptor, and ligating properties. These were recently discovered and further developed by the Plieger’s group, who were also able to somewhat optimize the initial Staab’s approach [11–14]. At the same time, we showed that its acenaphthene analog **5** can be synthesized in a single step in 55–60% yield directly from diamine **4** [15]. This result is contrary to the initial report [16]. Compound **5** still has the properties of a rather strong heterocyclic base, having higher basicity than “proton sponge” (Scheme 1) [15]. Although the physicochemical properties of acenaphthene **5**, including structure, protonation, and luminescence, have recently been studied by us in sufficient detail [15], nothing is known so far about the possibility of chemical modification of this molecule. It should be emphasized that the presence of a dimethylene moiety in the *peri*-positions of the naphthalene system will not only make molecule **5** (and derivatives) more rigid and flat when compared to compound **3** but it will also affect its reactivity and the sites of functionalization. This work is devoted to the clarification of this circumstance with substitution and elimination reactions chosen as the key transformations. The effect of functional groups on the further chemistry and basicity of the newly synthesized derivatives is also considered.

## Results and Discussion

### Amination, dehydrogenation, and supramolecular aggregation

**Figure 1:**
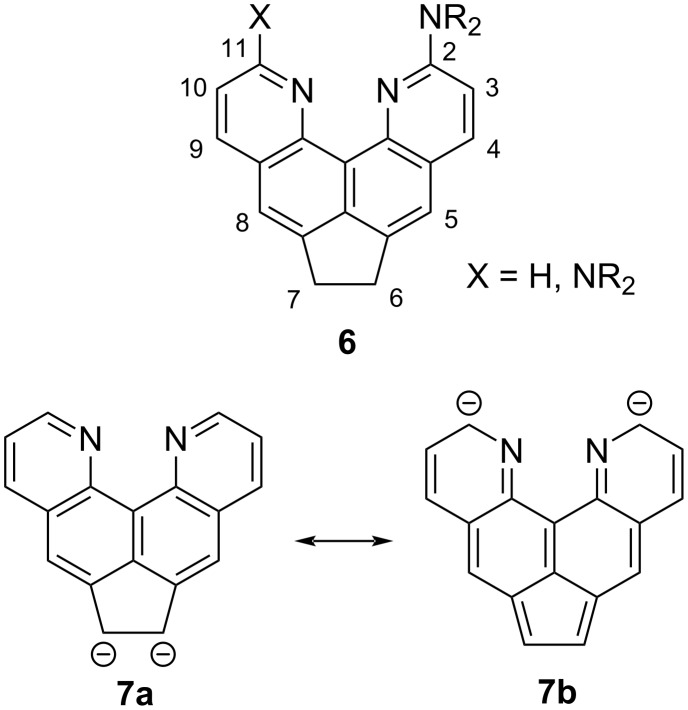
Suggested amination products **6** and two resonance forms of dianion **7**.

Direct amination of quinoquinoline **5** could potentially lead to 2(11)-substituted amines **6** (Figure 1), the basicity of which must obviously be higher than that of the starting heterocycle **5**. To this end, we conducted a series of experiments on its oxidative amination by varying the reaction conditions (time, temperature, addition of *n*-butyllithium to increase the nucleophilicity of amines), and reagents (amine, oxidizing agent) (for details and literature sources, see Supporting Information File 1, Table S1).

In most experiments, only starting compound **5** was isolated almost quantitatively from the reaction mixture. Azaarene **5** proved to be resistant to the action of reagents even in the presence of KMnO_4_ at 100 °C. The variation of the nucleophile did not give positive results either. For example, the replacement of dimethylamine with higher-boiling piperidine and *n*-butylamine or using liquid ammonia, which is highly reactive in such transformations, dipyridoacenaphthene **5** still returned unchanged, although quinoline and its derivatives are easily aminated under the same conditions [17]. No interaction occurred under the conditions of the Chichibabin reaction in an attempt to aminate compound **5** with sodium amide in *N*,*N*-dimethylaniline at 140–155 °C for an hour. Thus, quinoquinoline **5**, despite the presence of two pyridine nitrogen atoms, is extremely inert towards nucleophilic and oxidative amination reactions. Although molecule **5** does not explicitly contain deactivating electron-donating substituents such as alkoxy and dialkylamino (except for alkyl groups), the observed inertness may be associated with the increased basicity of the starting substrate and its structural organization (proximity of the two nitrogen atoms). The revealed inertness may also be rationalized via the ionization of the starting dipyridoacenaphthene system **5** to an anion or even dianion **7** (at least in equilibrium) under the action of an excess of nucleophiles as bases. Such dianions are characteristic of acenaphthene and have been repeatedly detected in subsequent transformations [18–19]. In our case, the CH-acidity of the CH_2_CH_2_ bridge should be even higher under the action of pyridine rings, and, if dianion **7** forms (resonance form **7b** will prevail in this case, Figure 1), it will be inactive to attack by nucleophiles. The behavior of acenaphthene **5** could be clarified further using its naphthalene analog **3**, which lacks benzylic CH_2_ protons, but there is no information in the literature about its activity/inactivity in amination reactions.

**Figure 2:**
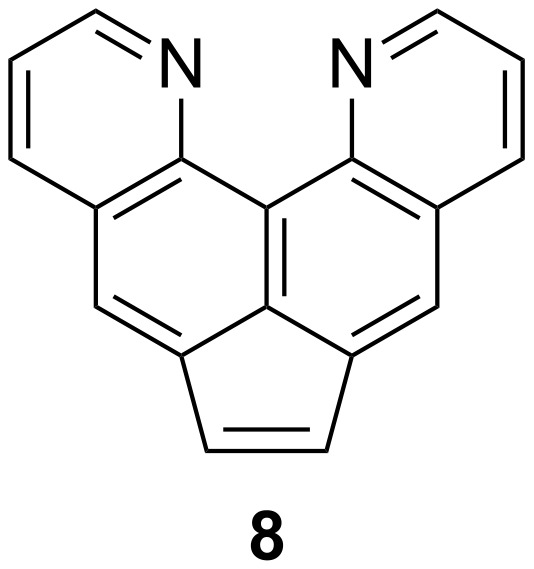
Targeted dipyridoacenaphthylene **8**.

It is known that acenaphthylenes are usually readily formed from acenaphthenes by dehydrogenation with chloranil, dichlorodicyanobenzoquinone (DDQ) or active MnO_2_ on reflux in toluene/xylene and other inert solvents. However, attempts to obtain acenaphthylene **8** (Figure 2) as a fully conjugated analog of acenaphthene **5** by classical methods were unsuccessful (Supporting Information File 1, Table S2). In all experiments, the starting compound remained unchanged or bound into a sparingly soluble precipitate even in high-boiling solvents such as *o*-dichlorobenzene or nitrobenzene.

**Scheme 2:**
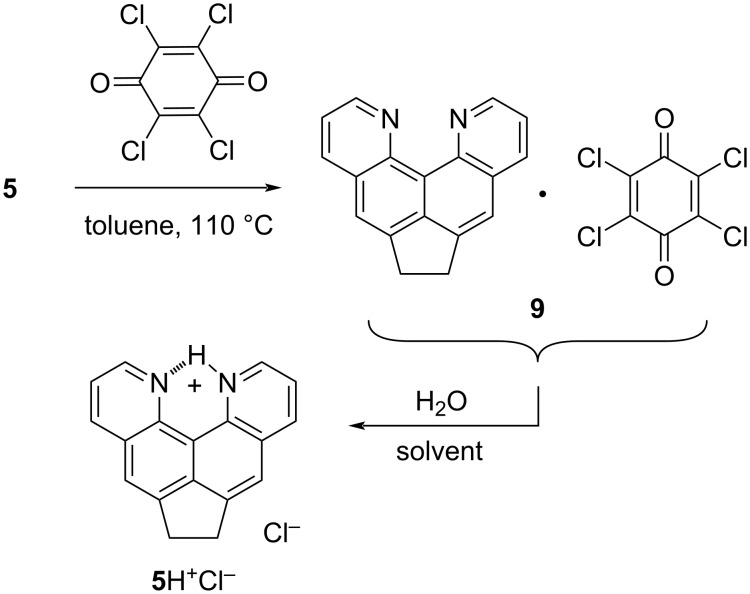
Formation of complex **9** and its slow hydrolytic degradation into protic salt **5·**HCl.

For example, quinoquinoline **5** reacts with chloranil upon heating in toluene to give a dark brown, high-melting product, in which, however, the methylene bridge remains unchanged. According to ^1^H NMR spectroscopy and combustion analysis data, this is complex **9** with a composition close to the ratio 1:1 (Scheme 2).

**Figure 3:**
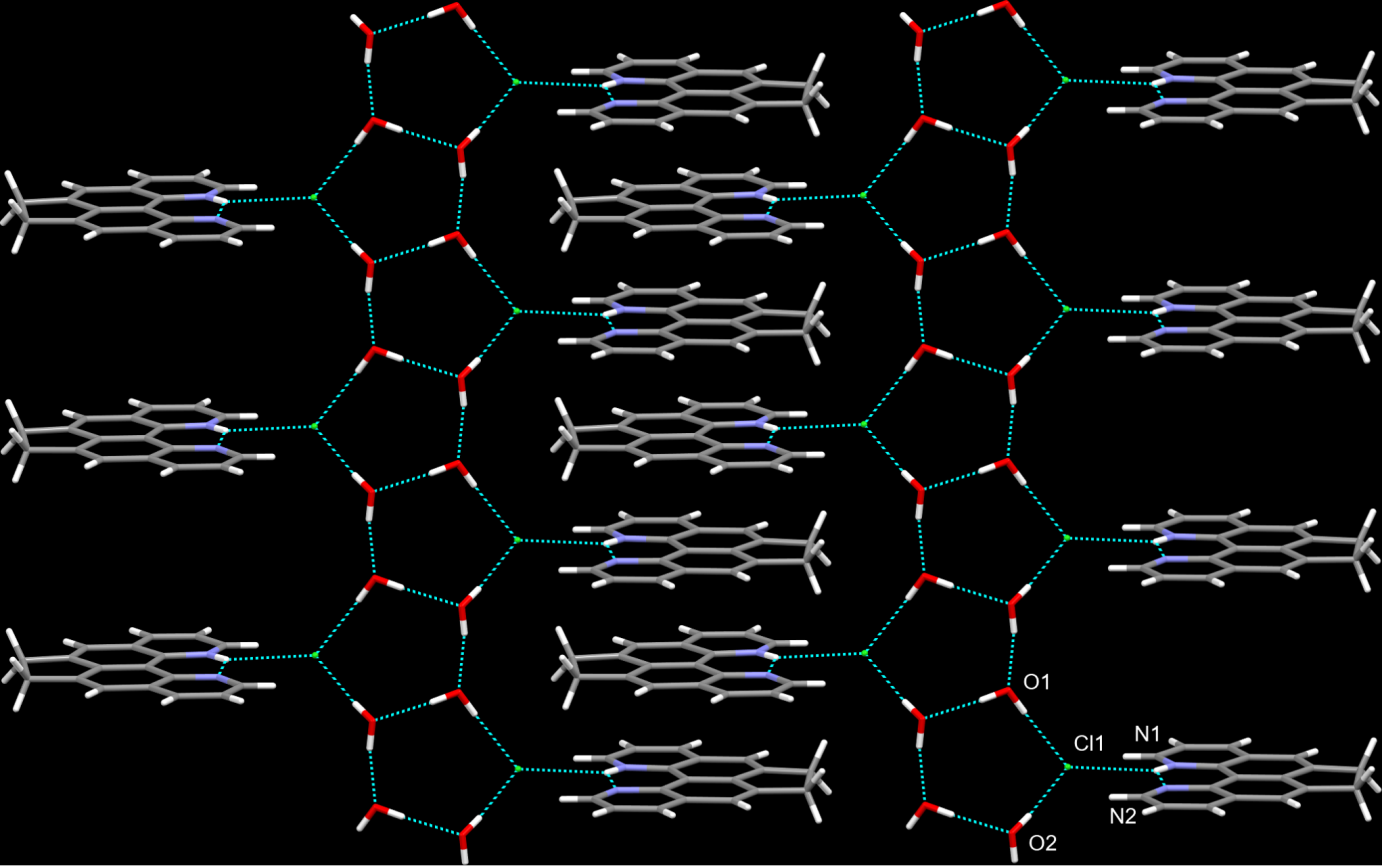
Molecular and crystal structure of salt **5·**HCl**·**2H_2_O is strongly dominated by severe H-bonding (blue dotted lines) and π-stacking (one preorganized layer along the *c*-axis is shown).

Complex **9** is insoluble in most organic solvents, but it turned out to be unstable in DMSO-*d*_6_ solution, as evidenced by monitoring its ^1^Н NMR spectra (Supporting Information File 1, Figure S1). Figure S1a shows the spectrum of the starting quinoline **5**, Figure S1b represents complex **9** 30 min after dissolution, while Figure S1c displays complex **9** three days later. Thus, in the spectrum of complex **9**, there is a distinct downfield shift of the signals of all protons in comparison with the same signals of base **5**. After three days, the shift noticeably increases simultaneously with the appearance of a signal at 17.5 ppm. The latter can be uniquely attributed to the signal of the proton chelated by the aza groups of the pyridine rings. Indeed, this spectrum almost completely coincides with the spectrum of protonated quinoquinoline, as shown in Figure S1d, depicting the spectrum of picrate **5**Н^+^PicO^−^, in the cationic part of which a similar intramolecular NHN hydrogen bond is realized [15]. To understand the structure of the resulting complex, we tried to grow its crystals from acetonitrile by co-evaporating solutions of quinoline **5** and chloranil at room temperature. Interestingly, in this case, hydrolytic degradation of chloranil also occurred during crystallization (base **5** could act as a catalyst for such degradation), because of which yellowish needles were obtained (neither **5** nor chloranil crystallize in this form), which turned out to be the hydrochloride dihydrate of compound **5**. As the XRD study of the crystals showed (Figure 3), the molecular and crystal structure of the isolated compound is strongly dominated, on the one hand, by intra- and intermolecular hydrogen bonds with the participation of N, Cl, and O heteroatoms (forming an endless slightly corrugated ribbon), and on the other hand, by π-stacking of the antiparallel protonated dipyridoacenaphthene fragments (two-dimensional dense stacks with an interplanar distance of 3.377 Å). Their combination is the main driving force behind the formation of the final supramolecular zipper structure.

**Figure 4:**
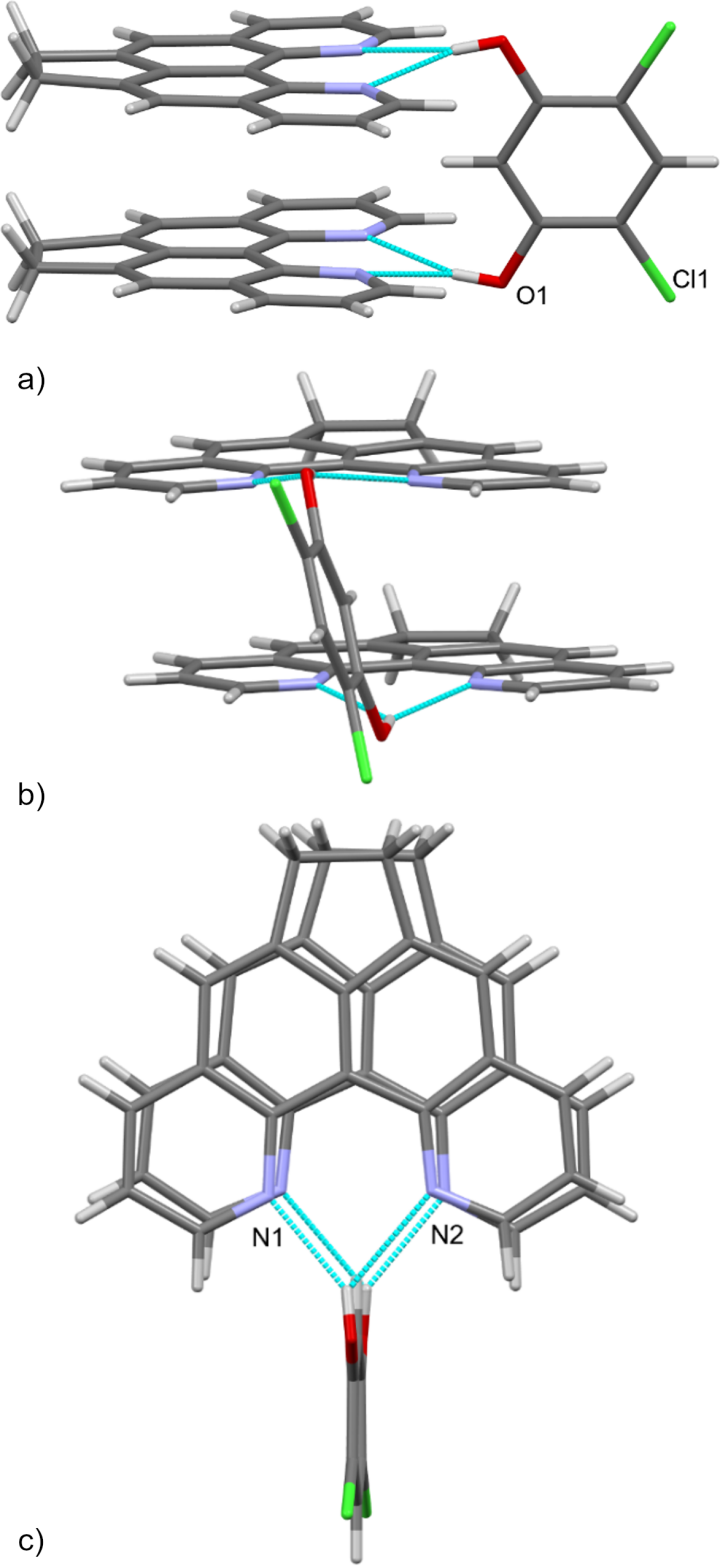
Selected images of the supramolecular organization of two molecules of base **5** held by 4,6-dichlororesorcinol in an almost parallel manner showing side view (а), view with the resorcinol molecule directed to the viewer (b), and view above the azaarene cycles showing their almost complete overlap (с). Again, π-stacking and H-bonding (blue dotted lines) strongly dominate in this structure.

Interestingly, the antiparallel orientation of the closely spaced cationic fragments of base **5** can be reversed to the opposite. This can be achieved with 4,6-dichlororesorcinol, a well-known molecular organizer and coordinating agent [20–21]. Thus, the joint crystallization of dipyridoacenaphthene **5** and 4,6-dichlororesorcinol in a 2:1 ratio leads to the formation of co-crystals, in which, as judged by the X-ray data, the supramolecular organization is again in action (Figure 4). Two molecules of the base are almost parallel to each other (the distance between the π-systems of two molecular planes is 3.551 Å with the divergence angle between them of only 1.33°) and are simultaneously connected by two bifurcated hydrogen bonds with the hydroxy groups of dichlororesorcinol.

**Figure 5:**
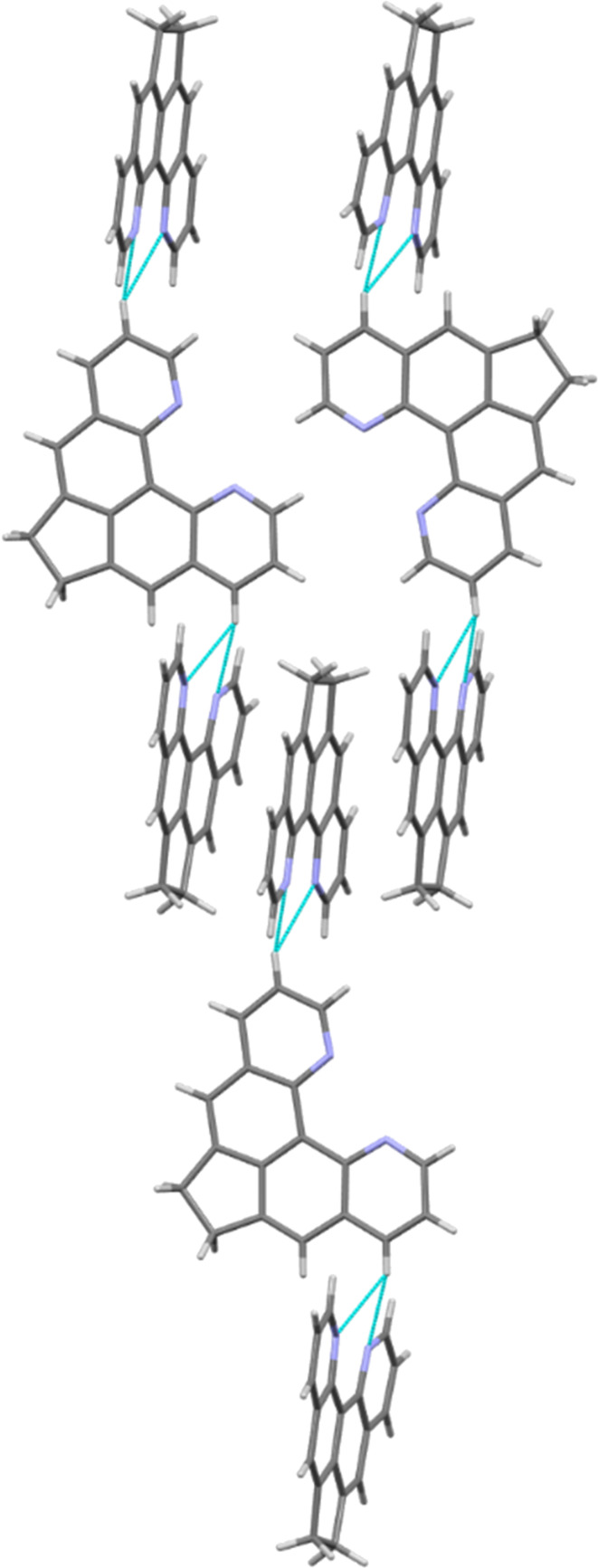
Fragment of the crystal packing of neutral dipyridoacenaphthene **5** showing self-association via multiple C–H^…^N contacts (blue dotted lines) between three independent molecules.

What would be the crystal structure of quinoquinoline **5** free of foreign particles? This is not an easy question to answer, since the superbasic nature of quinoquinolines, their planar structure, and very easy coordination to acidic and electrophilic sites (including water [15,22] or the C–H bond of chloroform [11]) almost always lead to co-crystallization. For example, there is no such crystallographic information for quinoquinoline **3** itself. In the present work, we succeeded in filling this gap by growing crystals of base **5** from pure acetonitrile. It turned out that molecule **5** is capable of self-association through multiple C–H^…^N–H-bond-like contacts involving pyridyl C(3)H and C(4)H protons (Figure 5). These intermolecular contacts, whose value lies in the range of 2.51–2.61 Å, strongly resemble the bifurcated hydrogen bonds so characteristic of base **5**, additionally reinforced by π-stacking between the terminal components in each H-associated triad (the shortest distance between the antiparallel π-systems of two molecular planes here is 3.353 Å).

Interestingly, as the acidity of the neighboring component in the crystal structure and the degree of proton transfer from it to the nitrogen atoms of quinoquinoline **5** decrease, the internitrogen distance regularly increases (N^…^N, Å): 2.709 (HCl), 2.808 (4,6-dichlororesorcinol), 2.813–2.835 (base **5**). At the same time, in contrast to quinoquinoline **3**, which sometimes adopts a twisted shape [11,13], molecule **5** each time remains almost flat.

### Nitration, nucleophilic methoxylation, and basicity measurements

**Scheme 3:**
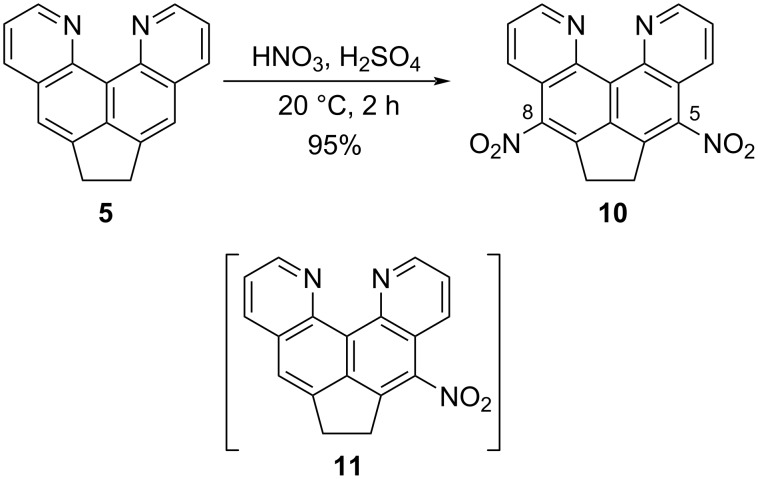
Dinitration of compound **5** and the initially assumed admixture **11**.

The nitration reaction of compound **5** should proceed in the same way as in other quinolines, at the benzene ring, and the resulting nitro compounds could potentially be subjected to further transformations, including nucleophilic substitution of nitro groups. Indeed, under the action of a small excess of the nitrating mixture, dipyridoacenaphthene **5** undergoes double nitration at positions 5 and 8 already at room temperature (Scheme 3). The overall yield of the main product **10** turned out to be high, but the substance contained a hard-to-separate impurity in an amount of up to 12%, to which, judged by the high-field position of the signals in the corresponding proton spectrum, was assigned the structure of the intermediate mononitro derivative **11** (Supporting Information File 1, Figure S2).

**Scheme 4:**
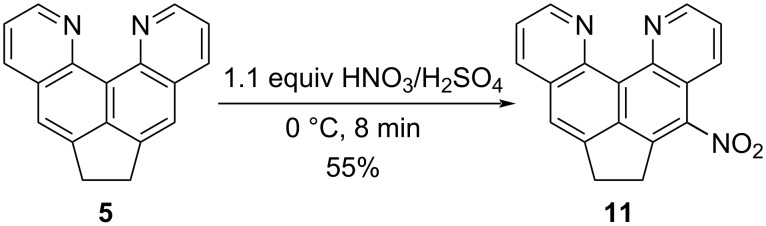
Mononitration of compound **5**.

Hoping to avoid formation of the “mononitro derivative” impurity, we increased the reaction time, the amount and composition of the nitrating mixture, and the temperature, but according to the results of ^1^H NMR spectra and TLC analysis, the second component was still present. This “permanent impurity” cannot be separated by chromatography or recrystallization, but it can be eliminated to some extent by washing the nitration products with hot chloroform, in which the impurity is slightly better soluble. This, however, is associated with losses of the main substance as well (Supporting Information File 1, Figure S3). Then, we decided to synthesize the mononitro derivative **11** purposefully by the action of one equivalent of the nitrating mixture. The reaction proceeded surprisingly well at 0 °C in several minutes with a good yield (Scheme 4). The spectral data fully confirmed the purity and asymmetric structure of product **11**. It should be emphasized that the isolation and purification of nitro compounds **10** and **11** is complicated by their low solubility in traditional organic solvents, sensitivity to sunlight (especially on adsorbents), and basic dipolar solvents (DMSO, for example, causes rather rapid degradation) [23]. Note that nitro derivatives **10** or **11** are not formed when base **5** is kept in nitric acid (25 °C, excess of 65% HNO_3_, 24 h) which returns the starting compound unchanged.

**Figure 6:**
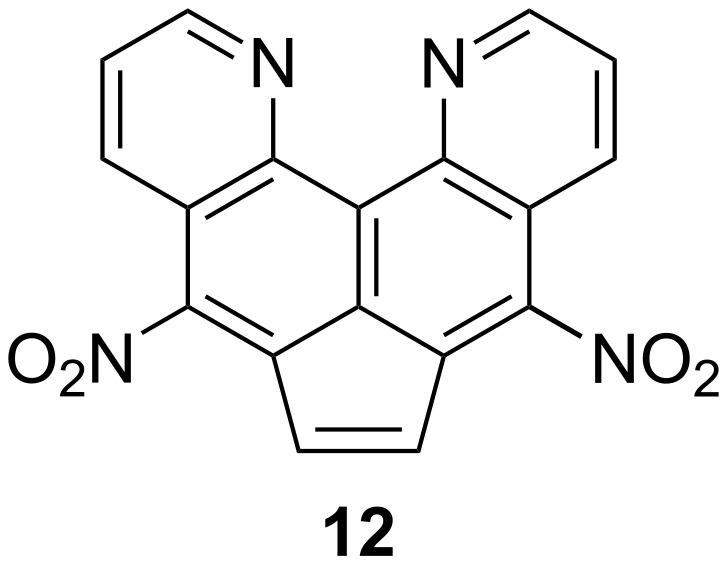
Structure of dinitroacenaphthylene **12**.

After that step, it became obvious that the second component formed in the dinitration reaction is not the mononitro derivative **11**, but acenaphthylene **12** (Figure 6).

**Scheme 5:**
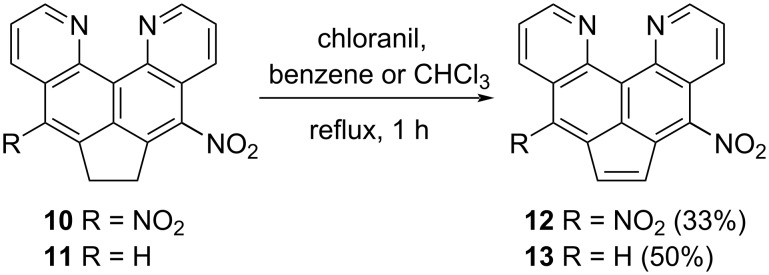
Dehydrogenation of compounds **10** and **11**.

To prove this hypothesis, as well as to compare the ease of dehydrogenation of dinitroacenaphthene **10** with respect to the initial substrate **5**, it was decided to carry out its oxidation to **12** by the traditional method – the action of chloranil in boiling benzene or chloroform (in the latter, the solubility of the components is somewhat better, although the temperature of the process decreases). At the end of the synthesis, the reaction mass was treated with a potassium hydroxide solution, and the oxidation product was isolated by chromatography. Nitroacenaphthylene **13** can also be obtained similarly (Scheme 5). Thus, although nitro groups usually hinder the dehydrogenation of acenaphthenes, in our case, the opposite trend is observed. We believe that the effect of nitro groups on the success of dehydrogenation here is associated with three circumstances: 1) a decrease in the basicity of the initial heterocycle **5** and, accordingly, a decrease in the degree of base-induced degradation of chloranil, 2) difficulties in the formation of molecular complexes due to the presence of 5(8) substituents, emerging from the plane of the π-system (see the previous section), and 3) acidification of the hydrogen atoms of the CH_2_ groups, which can facilitate their subsequent elimination.

The analysis of the ^1^Н NMR spectrum not only confirmed the structure of compound **12**, but also showed its identity to the sample obtained by the action of the nitrating mixture (Supporting Information File 1, Figures S2 and S7).

**Scheme 6:**
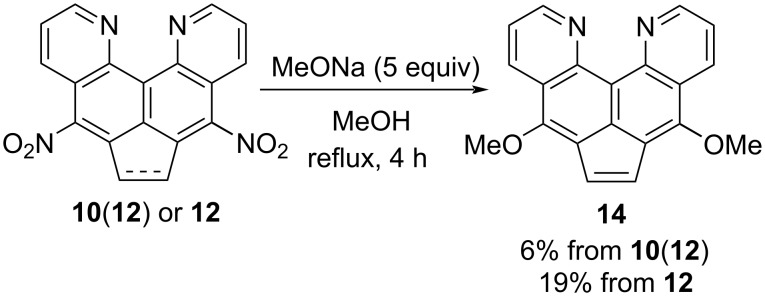
Nucleophilic methoxylation of compounds **10**(**12**).

Since the nitro groups in dinitroquinoquinoline **10** are formally in conjugated positions relative to the pyridine nitrogen atoms, they could potentially undergo a nucleophilic substitution. Indeed, upon boiling with an excess of sodium methoxide in methanol, the crude dinitration product **10**(**12**) gives up to 6% of a new substance with low mobility on sorbents and blue luminescence under UV light. Its spectral analysis confirmed the symmetrical structure with two methoxy groups, however, the CH_2_CH_2_ bridge was absent and the corresponding acenaphthylene **14** was obtained instead (Scheme 6).

Considering the low yield of dimethoxy product **14**, its potential source could be dinitroacenaphthylene **12**, which, as mentioned above, turned out to be a common impurity in compound **10**. The possibility of a double nucleophilic substitution without dehydrogenation was tested in a separate experiment with pure dinitro compound **12** taken as a starting material. Indeed, this variant produces the same dimethoxyacenaphthylene **14** in a noticeably higher yield (Scheme 6). In this case, the participation of the acenaphthylene derivative seems quite logical, since this should facilitate the S_N_Ar reaction and inhibit the formation of type **7** anions (under the action of methoxide as a base), which are inactive to subsequent nucleophilic attack.

**Figure 7:**
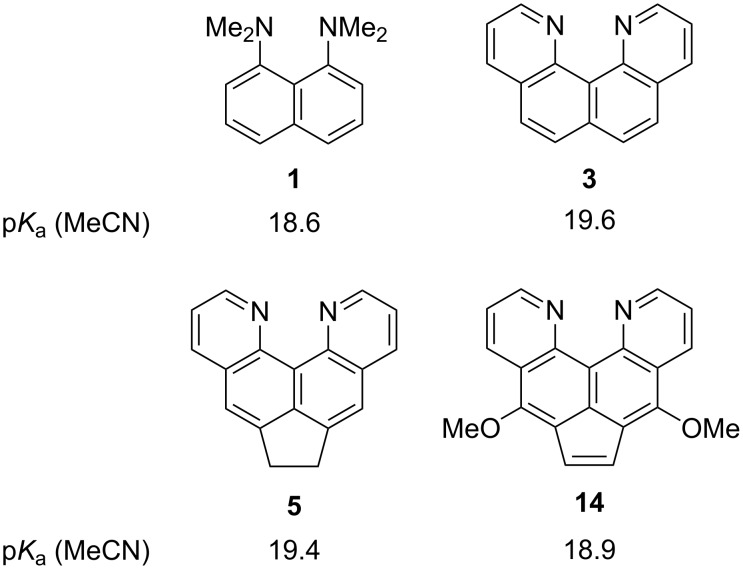
Basicity of key compounds in acetonitrile.

Dimethoxyacenaphthylene **14** is easily protonated, and its protic salt has been fully characterized as tetrafluoroborate **14**H^+^BF_4_^−^. The ^1^H NMR spectrum of this salt confirmed the symmetrical structure of the heterocyclic cation with a chelated intramolecular [NHN]^+^ bond, whose proton in CD_3_CN solution resonates at 17.22 ppm (Supporting Information File 1, Figure S13). This is a rather low value for chelate-type cations, but at the same time, it is quite logical, as molecule **14** contains a short CH=CH bridge, which increases the internitrogen distance and stretches (that is, weakens) the intramolecular hydrogen bond. For comparison, in the protonated cation of the starting diazine **5** in CD_3_CN, the chemical shift of the “acidic” proton is observed at 18.02 ppm [15]. Next, we evaluated the p*K*_a_ value of base **14** by a competitive method in acetonitrile (NMR transprotonation involving an equivalent amount of "proton sponge" **1** as a reference compound) [6]. Additionally, we measured the basicity of unsubstituted compound **5** in acetonitrile for the first time by the same method and the results are given in Figure 7; the p*K*_a_ values for compounds **1** and **3** are taken from references [24–25].

As can be seen, although diazine **14** turned out to be more basic than diamine **1**, the cumulative effect of all functional groups in this compound led to a slight drop in the p*K*_a_ value compared to quinoquinolines **3** and **5**, despite the presence of two electron-donating methoxy groups.

### Bromination and *tele*-elimination

**Scheme 7:**
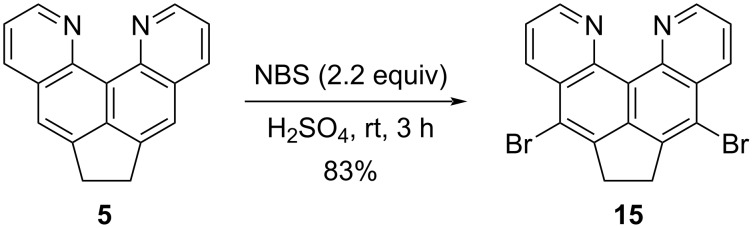
Electrophilic bromination of compound **5**.

As preliminary experiments showed, dipyridoacenaphthene **5** is not brominated by molecular bromine in chloroform or acetic acid. The action of the NBS–DMF system, previously proposed for the electrophilic bromination of alkylaromatic compounds [26], leaves substrate **5** unchanged at room temperature, and when heated to 75 °C for several days causes its gradual degradation. Obviously, in our case, the activating effect of the CH_2_CH_2_ fragment in the naphthalene part of molecule **5** is insufficient against the background of the presence of two pyridine rings in its structure. In this regard, we turned to concentrated sulfuric acid as a reaction medium and activator of NBS, as was previously shown by the example of a very successful bromination of 6-methylquinoline at position 5 with a preparative yield of 74% [27]. Indeed, under the new conditions, we obtained dibromo derivative **15** in high yield without heating and subsequent purification (Scheme 7).

The structure of compound **15** was confirmed by a combination of spectral methods, in particular, the disappearance of a singlet from H-5,8 protons at 7.8–7.9 ppm in the starting material **5** during functionalization (nitration, bromination), unambiguously indicates the occurrence of substituents precisely in these positions.

**Scheme 8:**
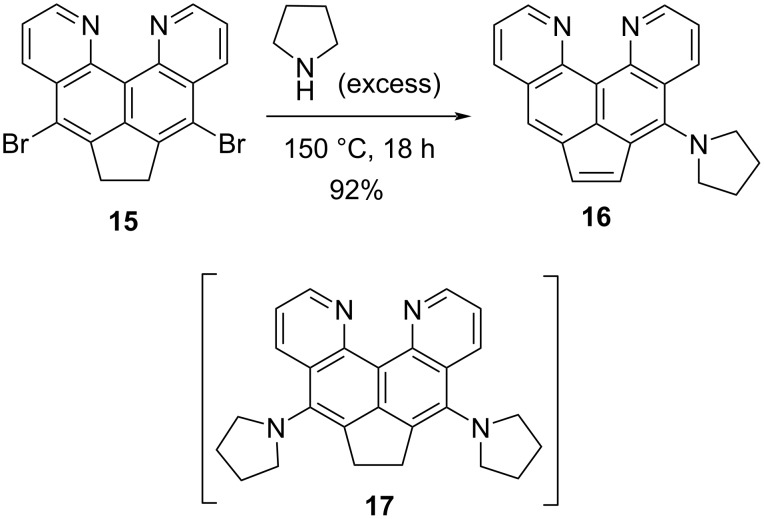
*tele*-Elimination upon interaction of dibromide **15** with pyrrolidine.

An attempt to dehydrogenate dibromide **15** was unsuccessful: the initial substrate remained unchanged after 2.5 hours of reflux in chloroform with one equivalent of chloranil (these conditions are practically similar to those used for the dehydrogenation of dinitro compound **10**). This result brings dibromide **15** closer in chemical properties to the parent compound **5**, which also cannot be converted into the corresponding acenaphthylene by direct dehydrogenation (see above). On the other hand, heating dibromide **15** with an excess of pyrrolidine for the purpose of nucleophilic substitution of the bromo-substituent led to a rather unexpected result. After cooling, dilution with water, basification, and extraction from the reaction mass, a single substance was isolated in almost quantitative yield, which turned out to be monosubstituted acenaphthylene **16**, rather than the expected disubstituted acenaphthene **17** (Scheme 8).

In addition to the spectral data confirming the composition and asymmetric structure of compound **16**, a clear sign of the emerging acenaphthylene system is its yellow-orange color, which distinguishes the UV-active (yellow-green luminescence) acenaphthylene **16** from the light-beige UV-inactive starting acenaphthene **15**. It is not possible to fix any intermediate products in this unusual transformation proceeding as *tele*-elimination with simultaneous nucleophilic substitution. Thus, carrying out the reaction under milder conditions (130 °C, 6 h) leads only to a mixture of **15** and **16** in a 45:55 ratio.

It should be recalled here that *tele*-elimination refers to the cleavage of molecular fragments located further than in the vicinal positions (at least three to five carbons between the hydrogen and bromine atoms in the case of **15** to give **16**). This rare transformation usually results in the formation of unsaturated products, often in the form of polyenes or alkynes [28]. The non-standard transition method found here in the acenaphthene–acenaphthylene pair is a previously unknown approach for the synthesis of acenaphthylenes based on *tele*-elimination (see also below).

**Scheme 9:**
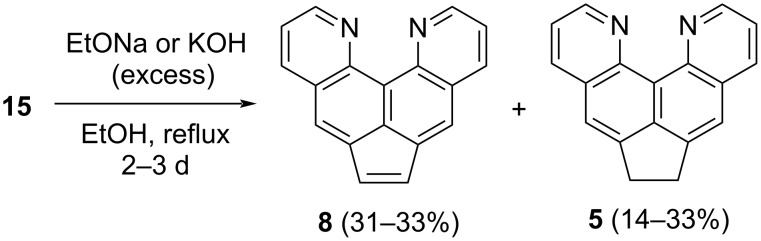
Interaction of dibromide **15** with anionic bases.

Changing the nucleophile to methoxide we tried to obtain an analog of compound **14** with the saturated CH_2_–CH_2_ bridge. While short-time heating leaves dibromide **15** mainly unchanged, refluxing for 3 days in the MeONa/MeOH system led to the formation of new compounds alongside the dibromo derivative **15**. Surprisingly, the ^1^H NMR spectrum showed the presence of acenaphthylene **8** and quinoline **5** as the major species in proportionate quantities. The use of sodium ethoxide in EtOH allowed us to carry out the reaction with full conversion in 2 days. Unfortunately, the admixtures and tarring formed in sufficient quantity made it difficult to purify compound **8**. At the same time, isolation of the new product **8** turned out to be more convenient on using the simple KOH/EtOH system. These conditions did not affect the yield of acenaphthylene **8** (Scheme 9). Compound **8** possesses fluorescence in solutions and the solid state both as the base and in the protonated form.

Thus, the unusual products of base treatment on dibromide **15** can be formally considered as a result of *tele*-elimination of bromine with the simultaneous shift of the two hydrogen atoms from the CH_2_CH_2_ bridge to positions 5(8) of the naphthalene system (formation of acenaphthylene **8**) or as a result of double protodebromination (giving acenaphthene **5**). Overall, the observed process resembles a redox transformation. Benzyl-type anions, which have hydride mobility and are formed in an alkaline environment from **15**, may act as a reducing agent here. We tried to stop this transformation (debromination of **15** under the action of hydroxides or alkoxides) at early stages to catch possible intermediates, but without success (for example, in the ^1^H NMR spectrum of the mixture there are no signals of methoxy groups when using MeONa). Additional experiments show that under the reaction conditions, quinoquinolines **5** and **8** do not transform into each other (prolonged boiling in an alcoholic KOH leaves them unchanged), and, therefore, are formed independently.

Next, the molecular structures of key molecules **5** and **8** in the form of their tetrafluoroborate salts were compared. For this, both compounds **5·**HBF_4_ and **8·**HBF_4_ were recrystallized from acetonitrile and subjected to XRD analysis under the same conditions. Selected data obtained are shown in Table 1. As can be seen, in both protonated quinoquinoline systems, an intramolecular hydrogen bond is realized (strongly asymmetric in crystals, but dynamically symmetric in solution), however, due to the noticeably larger internitrogen distance in cation **8**H^+^, the H bond in it is significantly weakened, as evidenced by a lower degree of deshielding of the chelated NH proton (cf. δ_NH_ values; see also data for protonated **14**) and a shorter counterion–NH proton contact (Table 1). Of course, the reason for this is the appearance in molecule **8** of a short CH=CH bridge, which enhances in-plane deformations of the entire molecular system [29]. As a result, the distance between the pyridine nitrogen atoms and, at the same time, the molecular rigidity naturally increase in the series **3** → **5** → **8** (“clothespin” effect) [30].

**Table 1:** Comparison of selected solid-state (XRD) and solution (^1^H NMR) parameters of dipyridoacenaphthene **5** (left) and dipyridoacenaphthylene **8** (right) taken as the monoprotonated tetrafluoroborates (the shortest distances between the N–H proton and the counterion are also shown).

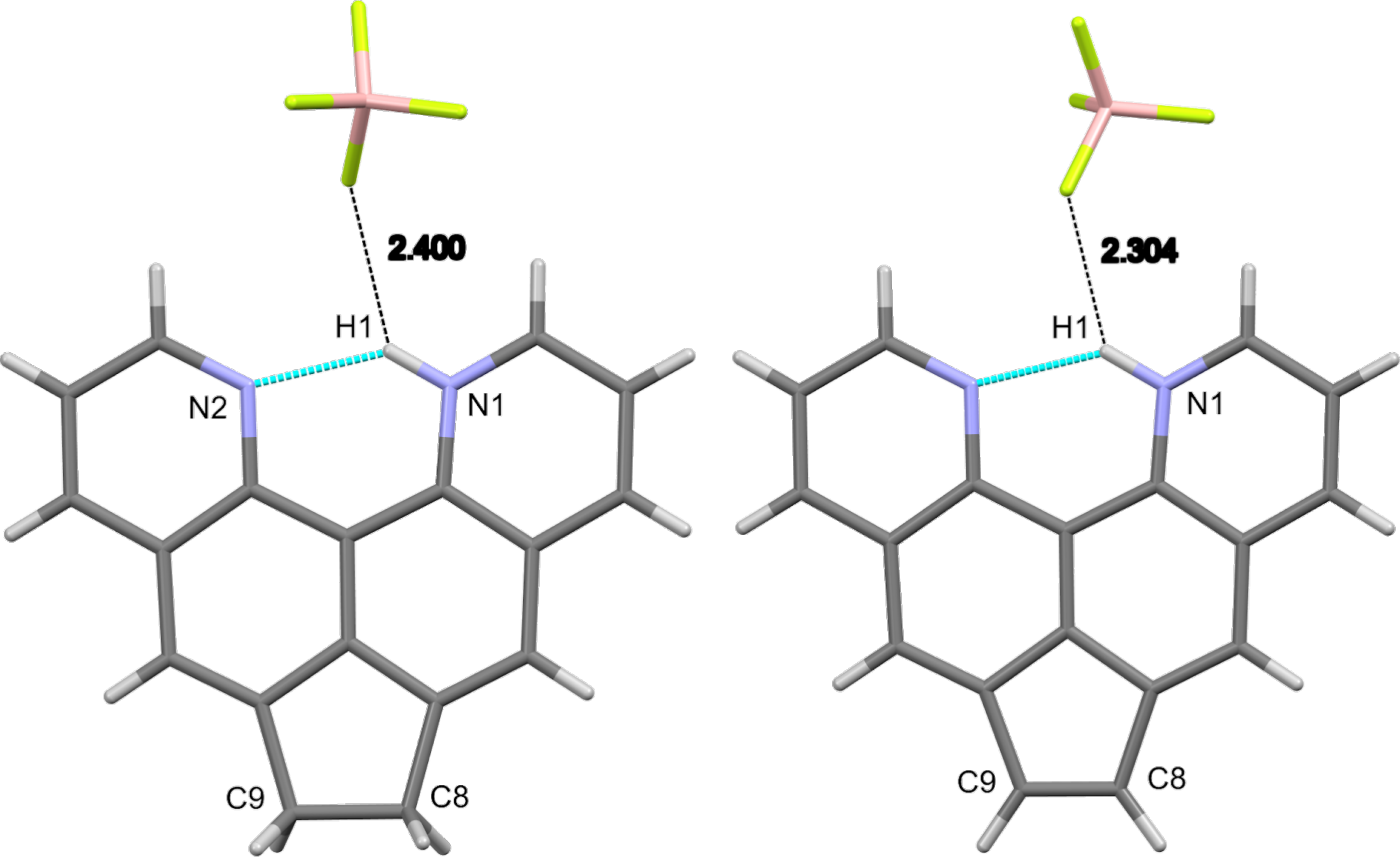		

Parameter	**5·**HBF_4_	**8·**HBF_4_

N^…^N distance, Å	2.697	2.712
N–H distance, Å	0.93	0.93
N^…^H distance, Å	1.94	1.96
C9–C8 distance, Å	1.554	1.354
∠NHN angle, °	137	136
δ_NH_, CD_3_CN	18.02	17.25
δ_H-2(11)_, CD_3_CN	9.19	9.06

The crystal packing patterns in salts **5·**HBF_4_ and **8·**HBF_4_ are quite similar. The main factor here continues to be the tendency of almost flat disk-shaped heterocyclic cations to π-stacking, leading to the formation of dense columns with anions in between (Supporting Information File 1, Figure S22). Flat dipyridoacenaphthylene cations **8**H^+^ give a denser packing, which, with an interplanar distance of only 3.328 Å, is the closest among all the studied compounds.

## Conclusion

Using single crystal XRD technique, dipyridoacenaphthene tetrafluoroborate, dipyridoacenaphthene chloride dihydrate, its 2:1 complex with 4,6-dichlororesorcinol, and neutral dipyridoacenaphthene as a self-associate were obtained and structurally characterized for the first time. The dominant feature of all crystal structures is the intramolecular NHN hydrogen bonding in combination with π-stacking of almost planar diazaarene fragments, leading to pronounced supramolecular aggregation. Although dipyridoacenaphthene does not undergo nucleophilic amination and dehydrogenation under a wide range of conditions, its 5(8)-nitro derivatives can be transformed under mild conditions into the corresponding acenaphthylene by the classical method using chloranil.

The potential activity of 5(8)-nitro groups in dipyridoacenaphthylene in nucleophilic substitution reactions was shown, and a 5,8-dimethoxy derivative containing both donor substituents and an acenaphthylene fragment was synthesized. Measurement of its basicity in acetonitrile medium showed that the combined effect of two methoxy groups and the acenaphthylene fragment is negative, leading to a decrease in basicity by 0.5 p*K*_a_ units compared to unsubstituted dipyridoacenaphthene, although 5,8-dimethoxydipyridoacenaphthylene is still more basic than the naphthalene "proton sponge".

A convenient and high yielding method was proposed to brominate dipyridoacenaphthene at positions 5 and 8 using a H_2_SO_4_/NBS system. The resulting dibromide turned out to be inert to dehydrogenation with chloranil, however, when heated with neutral (pyrrolidine) and anionic (NaOEt, KOH) bases, it can smoothly undergo *tele*-elimination, giving either functional derivatives or even unsubstituted and previously unknown dipyridoacenaphthylene. Since the discovered transformations are implemented in reasonable yields, they can be recommended as a new synthetic approach to acenaphthylene systems.

## Supporting Information

CCDC 2294253 (for 2(**5**)**·**(4,6-dichlororesorcinol)), 2294254 (for **5·**HBF_4_), 2294255 (for **8·**HBF_4_), 2294256 (for **5**), 2294257 (for **5·**HCl**·**2H_2_O) contain supporting crystallographic data for this paper. These data can be obtained free of charge from the Cambridge Crystallographic Data Center via https://www.ccdc.cam.ac.uk/data_request/cif.

File 1 Additional experimental and XRD information, synthetic procedures, copies of NMR spectral data for new compounds.

## Data Availability

All data that supports the findings of this study is available in the published article and/or the supporting information to this article.
