# Supplementary material for: Substitution reactions in the acenaphthene analog of quino[7,8-h]quinoline and an unusual synthesis of the corresponding acenaphthylenes by tele-elimination
Source: Beilstein J Org Chem. 2024 Feb 8;20:243–53. doi: 10.3762/bjoc.20.24 (PMC10862132; doi:10.3762/bjoc.20.24)
Supplement: File 1 — Additional experimental and XRD information, synthetic procedures, copies of NMR spectral data for new compounds. [file Beilstein_J_Org_Chem-20-243-s001.pdf]

BEILSTEIN JOURNAL OF ORGANIC CHEMISTRY

Supporting Information

for

Substitution reactions in the acenaphthene analog of quino[7,8-*h*]quinoline and an unusual synthesis of the corresponding acenaphthylenes by *tele*-elimination

Ekaterina V. Kolupaeva, Narek A. Dzhangiryan, Alexander F. Pozharskii, Oleg P. Demidov and Valery A. Ozeryanskii

Beilstein J. Org. Chem. **2024,** *20,* 243–253. doi:10.3762/bjoc.20.24

Additional experimental and XRD information, synthetic procedures, copies of NMR spectral data for new compounds

License and Terms: This is a supporting information file under the terms of the Creative Commons Attribution License (https://creativecommons.org/licenses/by/4.0). Please note that the reuse, redistribution and reproduction in particular requires that the author(s) and source are credited and that individual graphics may be subject to special legal provisions.
The license is subject to the *Beilstein Journal of Organic Chemistry* terms and conditions: (https://www.beilstein-journals.org/bjoc/terms)

Table of contents

Attempted amination of dipyrido[3,2-*e*:2′,3′-*h*]acenaphthene (Table S1) S2

Oxidative dehydrogenation of dipyrido[3,2-*e*:2′,3′-*h*]acenaphthene (Table S1)........ S3

Experimental data........ S4

Spectral data of pure and intermediate compounds (Figures S1–S21) S9

Crystal data and structure refinement for the studied compounds S21

Crystal packing in salts **5**·HBF_4 and **8**·HBF_4 (Figure S22) S22

Attempted amination of dipyrido[3,2-*e*:2′,3′-*h*]acenaphthene

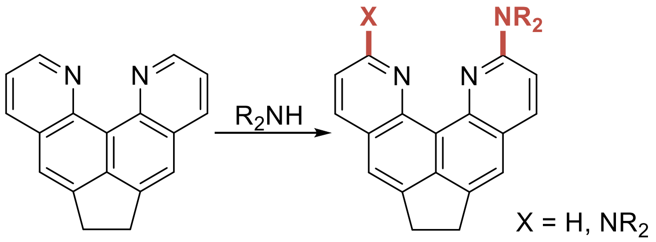

5 **6**

Table S1: Interaction of dipyrido[3,2-*e*:2′,3′-*h*]acenaphthene (**5**) with amines

Entry	Amine	Oxidant	Addition of *n*-BuLi	T, °C	Time (h)	Result of interaction
1	Me_2NH	$KMnO_4$	–	–15 ÷ –10	20	ND
2	Me_2NH	$KMnO_4$	+	–15 ÷ –10	24	ND
3	Me_2NH	$KMnO_4$	–	20	336	ND
4	Me_2NH	$AgPy_2MnO_4$	–	–15 ÷ –10	168	ND
5	Me_2NH	$AgPy_2MnO_4$	+	–15 ÷ –10	168	ND
6	Me_2NH	$AgPy_2MnO_4$	–	75–80 (sealed ampule)	3	ND
7	Me_2NH	$AgPy_2MnO_4$	–	95–100 (sealed ampule)	2.5	tarring
8	Me_2NH	$KMnO_4$	–	95–100 (sealed ampule)	0.5	ND
9	Piperidine	$KMnO_4$	–	20	144	ND
10	Piperidine	$KMnO_4$	+	20	120	ND
11	*n*-$BuNH_2$	$AgPy_2MnO_4$	–	20	24	ND
12	*n*-$BuNH_2$	$AgMnO_4$	–	20	2160	ND
13	NH_3	$KMnO_4$	–	–33	24	ND

ND – no product detected; only unreacted starting material was recovered.

For typical conditions and reagents used for amination and alkylamination of azaarenes (pyridines/quinolines) see the following works.[S1–S3]

[S1] Gulevskaya, A. V.; Pozharskii, A. F. The S_N^H-Amination of Heteroaromatic Compounds. In *Metal Free C-H Functionalization of Aromatics*; **2013**; 179–239. doi:10.1007/7081_2013_114

[S2] Gulevskaya, A. V.; Maes, B. U. W.; Meyers, C.; Herrebout, W. A.; van der Veken, B. J. *Eur. J. Org. Chem.* **2006**, 2006, 5305–5314. doi:10.1002/ejoc.200600573.

[S3] Wozniak, M.; van der Plas, H. C. *Acta Chem. Scand*. **1993**, 47, 95–101.

Oxidative dehydrogenation of dipyrido[3,2-*e*:2′,3′-*h*]acenaphthene

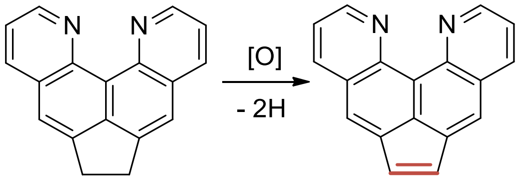

5 **8**

Table S2: Dehydrogenation of dipyrido[3,2-*e*:2′,3′-*h*]acenaphthene (**5**)

Entry	Oxidant	Solvent	*T*, °C	Time (h)	Result of interaction
1	chloranil	benzene	reflux	24	ND
2	chloranil	1,2-dichlorobenzene	reflux	12	ND
3	DDQ	benzene	reflux	24	ND
4	DDQ	$PhNO_2$	180	20	ND
5	MnO_2	benzene	reflux	12	ND
6	MnO_2	$PhNO_2$	180	14	ND

ND – no product detected.

Experimental data

^1H and ^{13}C NMR spectra were recorded on a Bruker DPX-250 (250 MHz) spectrometer with the solvent residual peaks as the internal standard. The HR–ESI mass spectra were obtained on a Bruker maXis spectrometer equipped with an electrospray ionization (ESI) source; methanol was used as the solvent. The instrument was operated in the positive mode using an *m*/*z* range of 50–1200. The capillary voltage of the ion source was set at 4000 V. The nebulizer gas pressure was 1.0 bar, and the drying gas flow was set to 4.0 L/min. Thin layer chromatography was carried out on Al_2O_3 and on silica gel (70–230 mesh, Aldrich). The progress of reactions and the purity of products were monitored by TLC on Al_2O_3; development with iodine vapor or UV-light. The melting points were measured in sealed capillaries and are uncorrected. The solvents were purified and dried by standard methods.

Crystal structure determination. XRD measurements were conducted with four-circle XtaLAB Synergy and SuperNova diffractometers, single source at home/near, HyPix3000 and AtlasS2 detectors. The structures were solved by direct methods and refined by the full-matrix least-squares against F2 in anisotropic (for nonhydrogen atoms) approximation. The C–H hydrogen atoms were placed in geometrically calculated positions and were refined in isotropic approximation in the riding model. The X–H (X = N, O) hydrogen atoms were found in the difference Fourier synthesis and refined in isotropic approximation. Atomic coordinates, bond lengths, bond angles, and thermal parameters have been deposited at the Cambridge Crystallographic Data Centre (CCDC).

Dipyridoacenaphthene 5 was prepared according to the published procedure.[S4] Light-yellow crystals, mp 230–232 °C (decomp.). The crystals, yellowish prisms, suitable for XRD measurements were obtained from acetonitrile.

Proton and molecular complexes (5·HX) were prepared by mixing equimolar amounts of base **5** and the corresponding acid (1 equivalent in the case of aqueous HBF_4 or 0.5 equivalents in the case of 4,6-dichlororesorcinol) in a minimum volume of acetonitrile followed by 5-fold dilution with Et_2O. The residue thus formed was washed with Et_2O, vacuum dried and recrystallized to give the desired salt almost quantitatively. The solvent was $CHCl_3$ in the case of 4,6-dichlororesorcinol followed by evaporation of the clear solution to dryness.

Tetrafluoroborate 5·HBF$_4$. Pale-yellow crystals, darken above 300 °C. For spectral and analytical data see ref.[S4] The crystals, yellowish needles, suitable for XRD measurements were obtained from acetonitrile.

Chloride dihydrate 5·HCl·2H$_2$O. The compound was occasionally obtained from a 1:1 mixture of **5** and chloranil on evaporation of its acetonitrile solution in air at ambient temperature. Small

[S4] Pozharskii, A. F.; Ozeryanskii, V. A.; Mikshiev, V. Y.; Chernyshev, A. V.; Metelitsa, A. V.; Antonov, A. S. *Org. Biomol. Chem.* **2019**, 17, 8221–8233.

pale-yellow crystals, darken above 290 °C. The crystals, pale-yellow needles, suitable for XRD measurements were obtained from acetonitrile.

Molecular complex 2(5)·(4,6-dichlororesorcinol). Light-yellow crystals, darken above 200 °C, decomp. 225–227 °C. The crystals, yellowish prisms, suitable for XRD measurements were obtained from a mixture of chloroform/acetonitrile.

Interaction of dipyridoacenaphthene 5 with chloranil. A mixture of compound **5** (70 mg, 0.27 mmol) and chloranil (67 mg, 0.27 mmol) in toluene (6 mL) was refluxed for 20 min. The residue formed was filtered, washed with toluene, and dried to give complex **9** (95 mg, 69%) as a dark brown solid with mp 243–245 °C. ^{1}H NMR (250 MHz, DMSO-d_6): δ = 9.28 (d, J = 3.2 Hz, 2H), 8.75 (d, J = 8.0 Hz, 2H), 8.09 (s, 2H), 7.91 (dd, J = 8.1, 4.4 Hz, 2H), 3.57 (s, 4H). Anal. Calcd for $C_{24}H_{12}Cl_4N_2O_2$: C, 57.40; H, 2.41; Cl, 28.24. Found: C, 54.48; H, 2.84; Cl, 26.24.

5,8-Dinitro-6,7-dihydropyrido[3',2':5,6]indeno[1,7-*gh*]quinoline (10). Dipyridoacenaphthene **5** (75 mg, 0.30 mmol) was dissolved in conc. H_2SO_4 (1.5 mL). The solution was cooled to 0 °C, 65% HNO_3 (1.0 mL, 15 mmol) was added within a minute, and the reaction mixture was stirred at rt for 2 h. The resulting mixture was poured onto crushed ice and basified with conc. aqueous ammonia solution to pH > 7. The yellow-colored residue was filtered and washed with distilled H_2O (3 × 2 mL) to give crude product **10** (99 mg, 95%). Further purification was carried out by dissolving compound **10** in boiling $CHCl_3$ (≈ 9 mL) and filtering. The residue was additionally washed with $CHCl_3$ (2 × 2 mL). The solvent was evaporated to afford **10** (60 mg, 58%) as a light-yellow, light sensitive solid which decomposes at 191–192 °C. ^{1}H NMR (250 MHz, $CDCl_3$): δ = 9.51 (dd, J = 4.2, 1.8 Hz, 2H), 8.84 (dd, J = 8.7, 1.8 Hz, 2H), 7.83 (dd, J = 8.7, 4.2 Hz, 2H), 3.92 (s, 4H). ^{13}C{^{1}H} NMR (62.9 MHz, $CDCl_3$): δ = 151.4, 146.0, 142.0, 141.4, 139.0, 131.9, 129.0, 123.4, 123.3, 30.5. HRMS (ESI): m/z $[M + H]^+$ calcd for $C_{18}H_{11}N_4O_4^+$: 347.0775; found: 347.0771.

5-Nitro-6,7-dihydropyrido[3',2':5,6]indeno[1,7-*gh*]quinoline (11). Dipyridoacenaphthene **5** (25 mg, 0.10 mmol) was dissolved in conc. H_2SO_4 (0.5 mL). The solution was cooled to 0 °C, 65% HNO_3 (7.5 µL, 0.11 mmol) was added, and the reaction mixture was stirred for 8 min. The resulting mixture was poured onto crushed ice and basified with conc. aqueous ammonia solution to pH > 7. The yellow-colored residue was filtered and washed with distilled H_2O (3 × 2 mL). The crude product was dissolved in boiling $CHCl_3$ (≈ 3 mL) and filtering. The residue was additionally washed with $CHCl_3$ (2 × 1 mL). The solvent was evaporated to give **11** (16 mg, 55%) as a yellow, light sensitive solid which decomposes at 170–172 °C. ^{1}H NMR (250 MHz, $CDCl_3$): δ = 9.46–9.41 (m, 2H), 8.80 (dd, J = 8.7, 1.8 Hz, 2H), 8.34 (dd, J = 8.2, 1.9 Hz, 2H), 7.93 (br s, 1H), 7.72 (dd, J = 8.7, 4.3 Hz, 1H), 7.66 (dd, J = 8.2, 4.3 Hz, 1H), 3.88–3.83 (m, 2H), 3.65–3.60 (m, 2H). ^{13}C{^{1}H} NMR (62.9 MHz, $CDCl_3$): δ = 150.6 (2C), 146.7, 145.4, 144.4 (2C), 141.9, 140.4, 136.8,

131.6, 131.4, 126.7, 124.0, 122.07, 122.05, 121.8, 30.8, 29.5. HRMS (ESI): m/z $[M + H]^+$ calcd for $C_{18}H_{12}N_3O_2^+$: 302.0924; found: 302.0921.

5,8-Dinitropyrido[3',2':5,6]indeno[1,7-*gh*]quinoline (12). *Route 1.* A mixture of compound **10** (20 mg, 0.06 mmol) and chloranil (25 mg, 0.10 mmol) in benzene (8 mL) was refluxed for 1 h in darkness. The hot resulting mixture was filtered off to remove admixtures. The solution was washed with 10% aqueous KOH (6 × 10 mL) to remove chloranil and its derivatives. The solvent was evaporated, and the crude product was purified by PTLC (silica gel, $CHCl_3$) to give **12** (4 mg, 19%) as a bright-yellow solid which decomposes at 223–225 °C. ^1H NMR (250 MHz, $CDCl_3$): 9.49 (dd, *J* = 4.4, 1.8 Hz, 2H), 8.77 (dd, *J* = 8.7, 1.8 Hz, 2H), 7.82 (dd, *J* = 8.7, 4.3 Hz, 2H), 7.57 (s, 2H). $^{13}C\{^1H\}$ NMR (62.9 MHz, $CDCl_3$): δ = 152.0, 135.1, 133.8, 133.6, 123.4 (some carbons are missed due to low solubility and instability of the substance in solutions). HRMS (ESI): m/z $[M + H]^+$ calcd for $C_{18}H_9N_4O_4^+$: 345.0618; found: 345.0623.

Route 2. A mixture of compound **10** (20 mg, 0.06 mmol) and chloranil (25 mg, 0.10 mmol) in $CHCl_3$ (5 mL) was refluxed for 1 h in darkness. The solvent was evaporated, and the crude product was purified by column chromatography (Al_2O_3, $CHCl_3$) to give **12** (7 mg, 33%) with the properties identical to those of the sample prepared by *Route 1*.

5-Nitropyrido[3',2':5,6]indeno[1,7-*gh*]quinoline (13). A mixture of compound **11** (14 mg, 0.04 mmol) and chloranil (12 mg, 0.05 mmol) in benzene (8 mL) was refluxed for 1 h in darkness. The hot resulting mixture was filtered off to remove admixtures. The solution was washed with 10% aqueous KOH (6 × 10 mL) to remove chloranil and its derivatives. The solvent was evaporated to give **13** (6 mg, 50%) as a yellow solid which decomposes at 195–197 °C. ^1H NMR (250 MHz, $CDCl_3$): 9.46 (dd, *J* = 4.4, 1.8 Hz, 1H), 9.39 (dd, *J* = 4.5, 1.9 Hz, 1H), 8.80 (dd, *J* = 8.7, 1.8 Hz, 1H), 8.38 (dd, *J* = 8.1, 1.9 Hz, 1H), 8.09 (s, 1H), 7.75 (dd, *J* = 8.7, 4.3 Hz. 1H), 7.65 (dd, *J* = 8.1, 4.4 Hz, 1H), 7.43 (s, 2H). $^{13}C\{^1H\}$ NMR (62.9 MHz, $CDCl_3$): δ = 151.34, 151.25, 147.8, 147.7, 138.5, 138.0, 136.3, 134.0, 133.1, 132.9, 131.3, 131.1, 129.4, 127.4, 125.1, 122.1 (2C), 121.6. HRMS (ESI): m/z $[M + H]^+$ calcd for $C_{18}H_{10}N_3O_2^+$: 300.0768; found: 300.0765.

5,8-Dimethoxypyrido[3',2':5,6]indeno[1,7-*gh*]quinoline (14). Compound **12** (34 mg, 0.10 mmol) was added to a solution of sodium (12 mg, 0.52 mmol) in MeOH (5 mL). The reaction mixture was refluxed for 4 h. The solvent was evaporated, and the crude product was purified by column chromatography (Al_2O_3, $CHCl_3$) to give **14** (6 mg, 19%) as a light-yellow solid (its solutions possess blue fluorescence under UV light), which decomposes at 216–217 °C. ^1H NMR (250 MHz, $CDCl_3$): 9.37 (dd, *J* = 4.3, 1.9 Hz, 2H), 8.90 (dd, *J* = 8.4, 1.9 Hz, 2H), 7.62 (s, 2H), 7.55 (dd, *J* = 8.4, 4.3 Hz, 2H), 4.50 (s, 6H). $^{13}C\{^1H\}$ NMR (62.9 MHz, $CDCl_3$): δ = 151.0, 150.8, 148.1, 138.2, 132.6, 126.1, 122.8, 122.7, 119.7, 118.0, 60.8. HRMS (ESI): m/z $[M + H]^+$ calcd for $C_{20}H_{15}N_2O_2^+$: 315.1128; found: 315.1133.

5,8-Dimethoxypyrido[3',2':5,6]indeno[1,7-*gh*]quinoline hydrogen tetrafluoroborate (14·HBF$_4$). Compound **14** (10 mg, 0.03 mmol) was partially dissolved in MeCN (1 mL) and treated with 40% aqueous HBF$_4$ (6.0 µL, 0.03 mmol) resulting in a homogeneous solution. The solvent was evaporated, the residue was washed with Et$_2$O (2 × 2 mL) and vacuum-dried to give **14**·HBF$_4$ (5 mg, 42%) as a dark-yellow solid with mp 284–285 °C. ^{1}H NMR (250 MHz, CD$_3$CN): 17.22 (s, 1H), 8.91–8.88 (m, 4H), 7.78 (dd, *J* = 8.7, 5.6 Hz, 2H), 7.46 (s, 2H), 4.42 (s, 6H).

5,8-Dibromo-6,7-dihydropyrido[3',2':5,6]indeno[1,7-*gh*]quinoline (15). Compound **5** (75 mg, 0.30 mmol) was dissolved in conc. H$_2$SO$_4$ (1.0 mL). NBS (118 mg, 0.66 mmol) was added to the solution and the reaction mixture was stirred at rt for 3 h. The resulting mixture was poured onto crushed ice, basified with solid KOH to pH 9–10, and extracted with CHCl$_3$ (6 × 10 mL). The solvent was removed to give pure **15** (103 mg, 83%) as a light-beige solid which decomposes at temperatures above 300 °C. ^{1}H NMR (250 MHz, CDCl$_3$): δ = 9.40 (dd, *J* = 4.3, 1.8 Hz, 2H), 8.80 (dd, *J* = 8.5, 1.8 Hz, 2H), 7.69 (dd, *J* = 8.5, 4.3 Hz, 2H), 3.54 (s, 4H). ^{13}C{^{1}H} NMR (62.9 MHz, CDCl$_3$): δ = 150.6, 146.9, 145.5, 141.0, 135.6, 128.7, 121.9, 118.4, 31.9. HRMS (ESI): m/z [M + H]$^+$ calcd for $C_{18}H_{11}Br_2N_2^+$: 414.9264; found: 414.9257.

5-(Pyrrolidin-1-yl)pyrido[3',2':5,6]indeno[1,7-*gh*]quinoline (16). A mixture of dibromide **15** (16 mg, 0.04 mmol) and pyrrolidine (1.8 mL) was heated in a sealed ampule for 18 h at 150 °C. Then, the reaction mixture was allowed to cool to rt, poured in H$_2$O (20 mL), basified with aqueous KOH to pH 10–11, and extracted with CH$_2$Cl$_2$ (3 × 7 mL). The crude product was purified by column chromatography (Al$_2$O$_3$, CHCl$_3$) to give **16** (12 mg, 92%) as a yellow-orange solid (its solutions possess yellow-green fluorescence under UV light) with mp 124–126 °C. ^{1}H NMR (250 MHz, CDCl$_3$): δ = 9.35 (dd, *J* = 4.1, 1.6 Hz, 1H), 9.29 (dd, *J* = 4.3, 1.8 Hz, 1H), 8.66 (dd, *J* = 8.5, 1.8 Hz, 1H), 8.39 (dd, *J* = 8.1, 1.8 Hz, 1H), 8.12 (s, 1H), 7.68 (d, *J* = 5.4 Hz, 1H), 7.54–7.44 (m, 2H), 7.17 (d, *J* = 5.4 Hz, 1H), 3.81–3.76 (m, 4H), 2.13–2.07 (m, 4H). ^{13}C{^{1}H} NMR (62.9 MHz, CDCl$_3$): δ = 150.1, 150.0, 149.9, 146.1, 146.0, 145.8, 137.7, 136.7, 135.8, 135.3, 131.9, 127.6, 125.8, 124.9, 123.4, 120.7, 119.4, 118.5, 54.8, 25.8. HRMS (ESI): m/z [M + H]$^+$ calcd for $C_{22}H_{18}N_3^+$: 324.1495; found: 324.1500.

Interaction of dibromide 15 with anionic bases. *Route 1*. Dibromide **15** (82 mg, 0.20 mmol) was added to a solution of sodium (345 mg, 15 mmol) in EtOH (12 mL). The reaction mixture was refluxed for 2 d. The resulting mixture was poured in H$_2$O (40 mL) and extracted with CHCl$_3$ (3 × 10 mL). The solvent was removed, and the crude products were purified by column chromatography (Al$_2$O$_3$, CH$_2$Cl$_2$) to give **8** (16 mg, 31%) followed by **5** (17 mg, 33%).

Route 2. Dibromide **15** (82 mg, 0.20 mmol) was added to a solution of KOH (1.12 g, 20 mmol) in EtOH (12 mL). The reaction mixture was refluxed for 3 d. The resulting mixture was poured in H$_2$O (40 mL) and extracted with CHCl$_3$ (3 × 10 mL). The solvent was removed, and the crude

products were purified by column chromatography (Al_2O_3, CH_2Cl_2) to give **8** (17 mg, 33%) followed by **5** (7 mg, 14%).

Pyrido[3',2':5,6]indeno[1,7-*gh*]quinoline (dipyrido[3,2-*e*:2′,3′-*h*]acenaphthylene) (8). Beige solid which possesses green fluorescence under UV light in solutions and solid state; mp 179–181 °C. ^{1}H NMR (250 MHz, $CDCl_3$): δ = 9.38 (dd, *J* = 4.4, 1.9 Hz, 2H), 8.38 (dd, *J* = 8.1, 1.9 Hz, 2H), 8.06 (s, 2H), 7.59 (dd, *J* = 8.1, 4.4 Hz, 2H), 7.30 (s, 2H). ^{13}C{^{1}H} NMR (62.9 MHz, $CDCl_3$): δ = 150.6, 148.0, 138.0, 134.6, 131.8, 130.1, 124.8, 122.9, 120.8. HRMS (ESI): m/z $[M + H]^+$ calcd for $C_{18}H_{11}N_2^+$: 255.0917; found: 255.0920.

Dipyridoacenaphthene 5. Light-yellow solid; mp, ^{1}H NMR, and other properties are consistent with the published data.[S4]

Pyrido[3',2':5,6]indeno[1,7-*gh*]quinoline hydrogen tetrafluoroborate (8·HBF$_4$). Compound **8** (8 mg, 0.03 mmol) was treated with 40% aqueous HBF_4 (6.0 µL, 0.03 mmol) in a minimum volume of MeCN (0.6 mL) followed by dilution with Et_2O (9 mL). The residue formed was washed with Et_2O (9 mL) and vacuum-dried to give **8**·HBF_4 (8 mg, 80%) as a yellow solid which darkens above 308–310 °C. Salt **8**·HBF_4 possesses green fluorescence under UV light in solutions and solid state. Crystals suitable for XRD analysis were obtained from MeCN. ^{1}H NMR (250 MHz, CD_3CN): 17.25 (s, 1H), 9.07–9.05 (m, 2H), 8.85 (dd, *J* = 8.2, 1.6 Hz, 2H), 8.15 (s, 2H), 7.96 (dd, *J* = 8.5, 5.0 Hz, 2H), 7.31 (s, 2H). ^{13}C{^{1}H} NMR (62.9 MHz, CD_3CN): δ = 146.2, 144.9, 143.8, 141.5, 139.0, 135.2, 133.5, 130.1, 125.3, 123.1.

Spectral data of pure and intermediate compounds

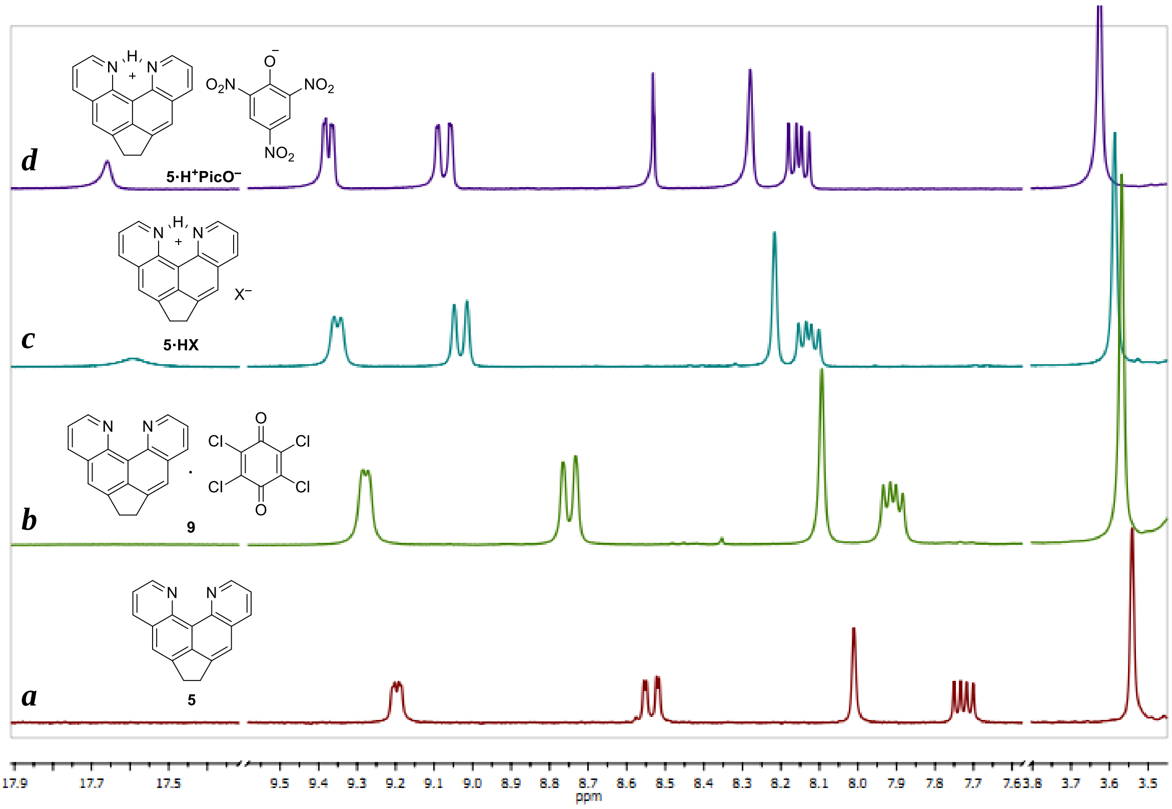

Figure S1: ^{1}H NMR spectra of complex **9**: ***a*** – pure quinoquinoline **5**; ***b*** – complex **9**, 30 min after dissolution; ***c*** – complex **9**, 3 d after dissolution; ***d*** – picrate **5**·H^{+}PicO^{-} (250 MHz, DMSO-d_6).

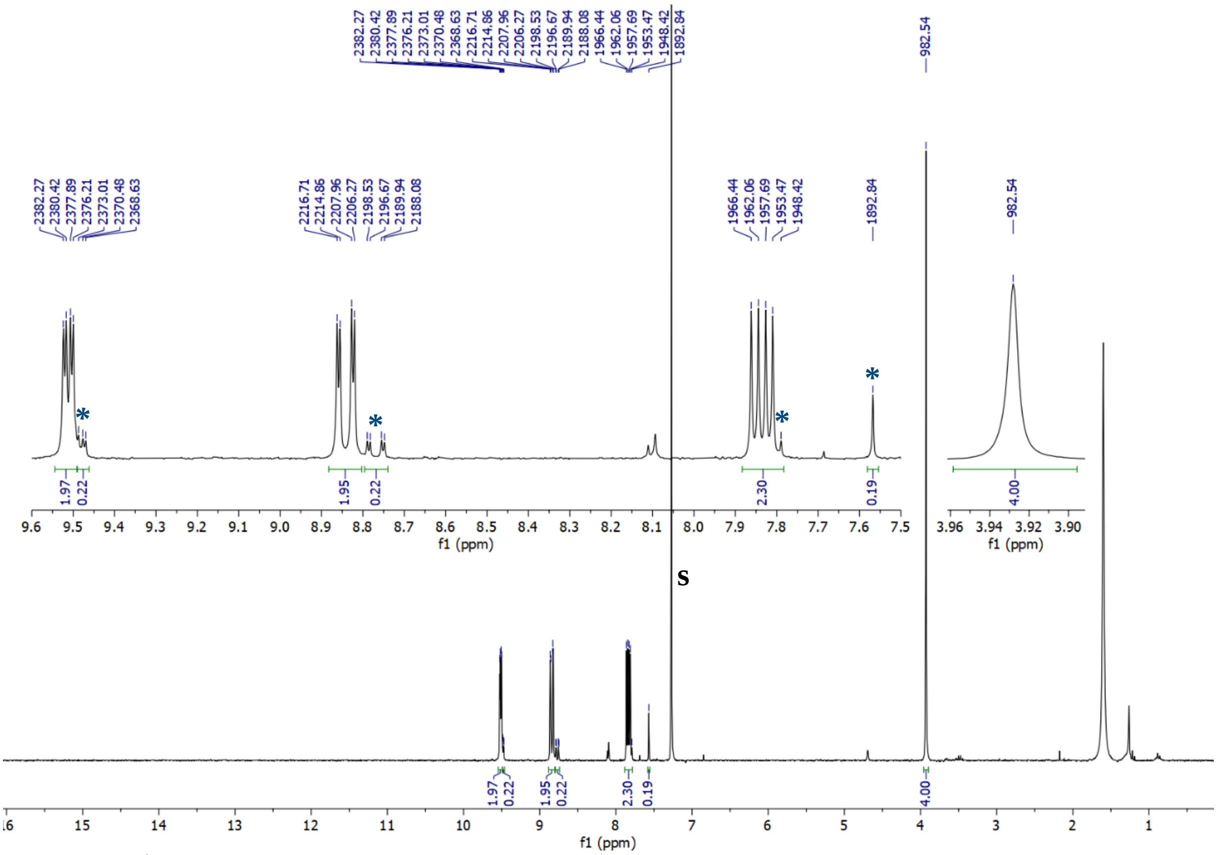

Figure S2: ^{1}H NMR spectrum of **10** and an “unknown” admixture (*) in a ~9:1 ratio (250 MHz, $CDCl_3$).

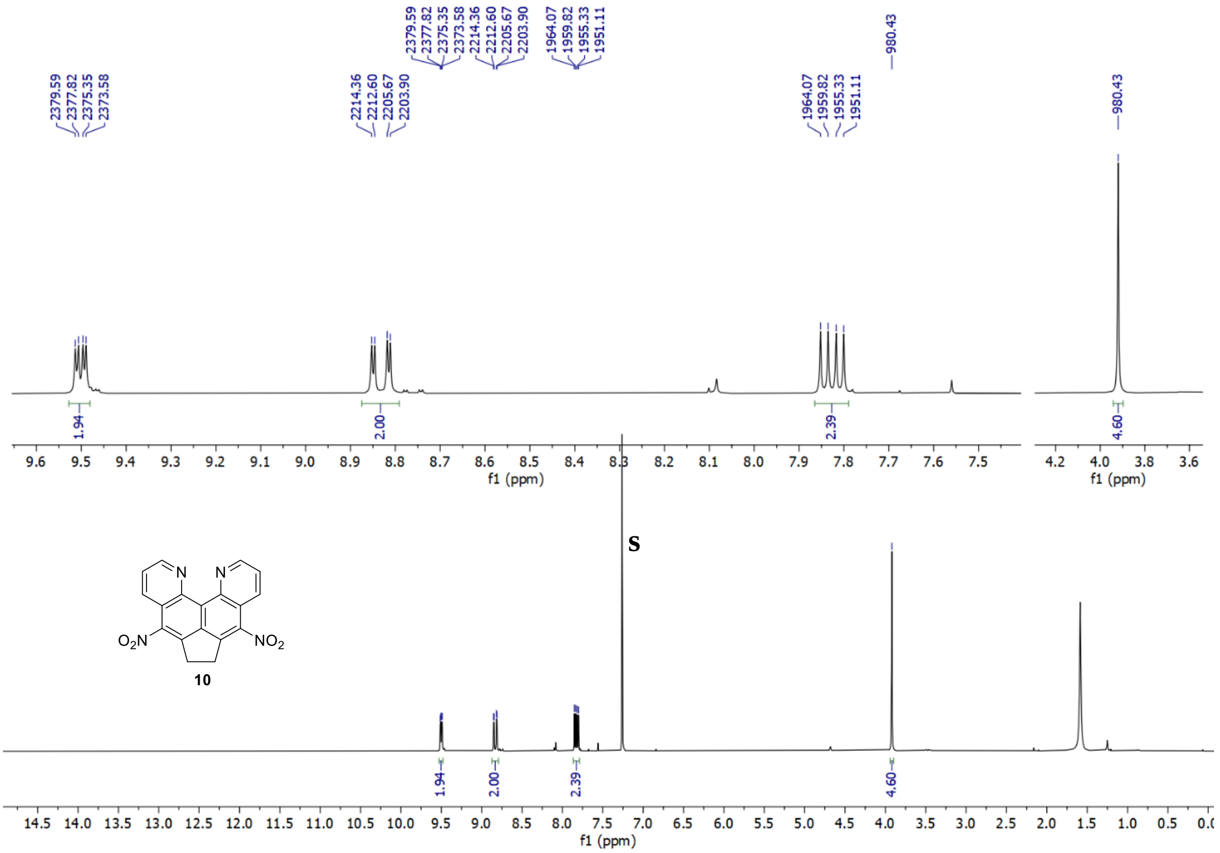

Figure S3: ^{1}H NMR spectrum of **10** (250 MHz, $CDCl_3$).

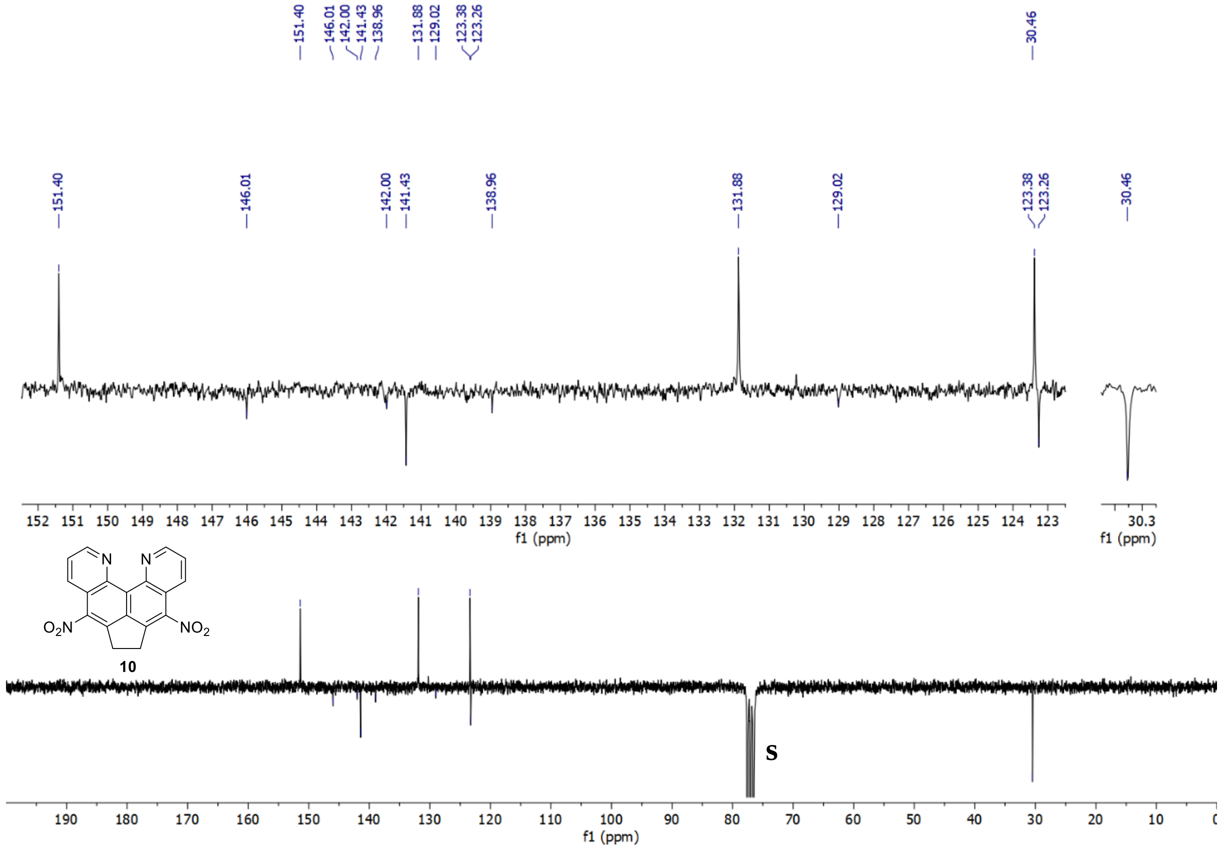

Figure S4: $^{13}C\{^{1}H\}$ APT-NMR spectrum of **10** (62.9 MHz, $CDCl_3$).

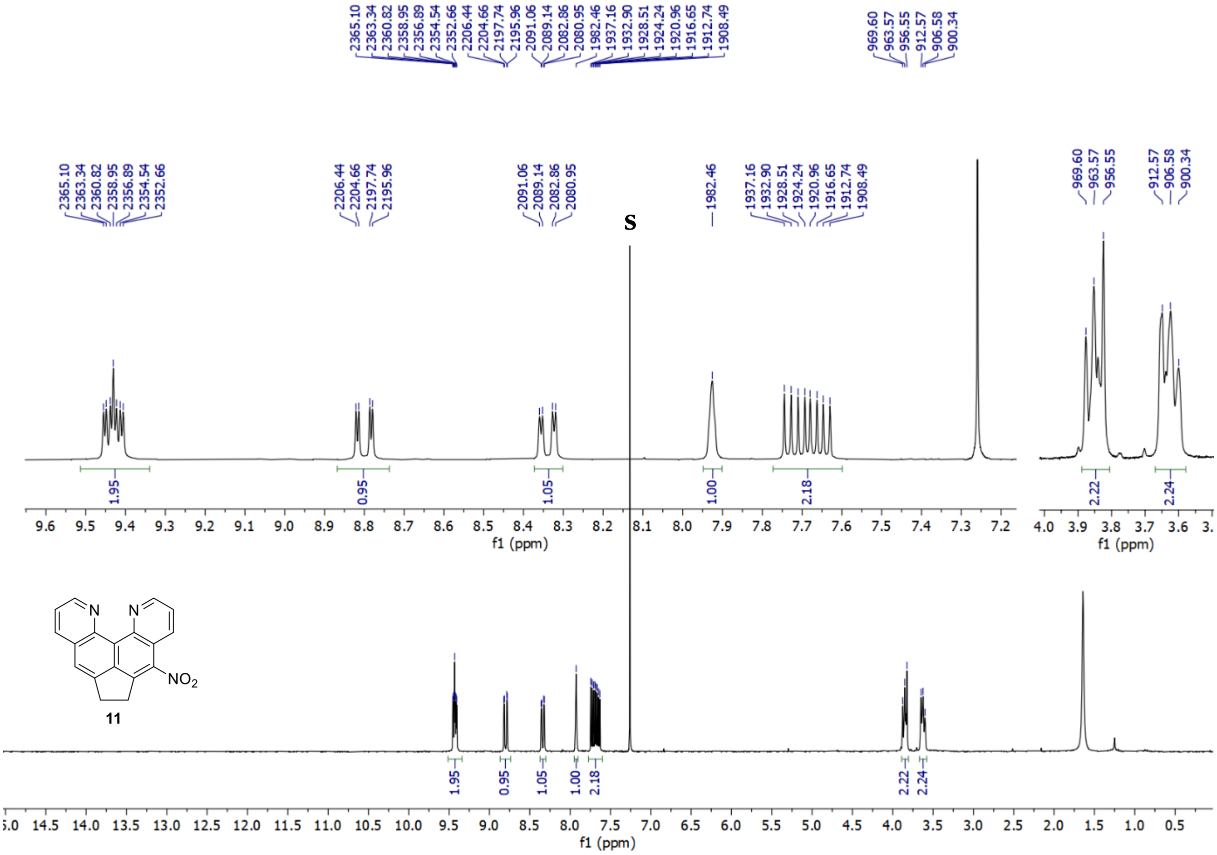

Figure S5: ^{1}H NMR spectrum of **11** (250 MHz, $CDCl_3$).

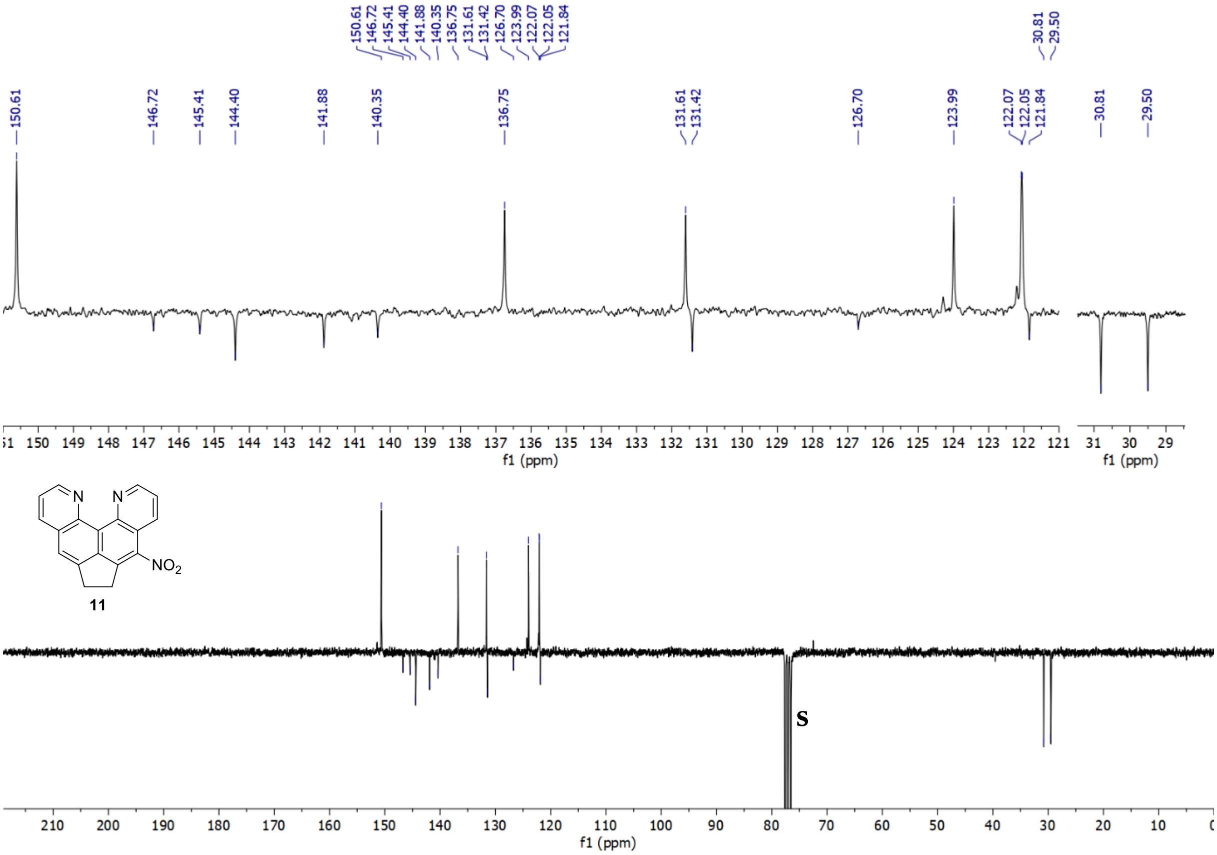

Figure S6: $^{13}C\{^{1}H\}$ APT-NMR spectrum of **11** (62.9 MHz, $CDCl_3$).

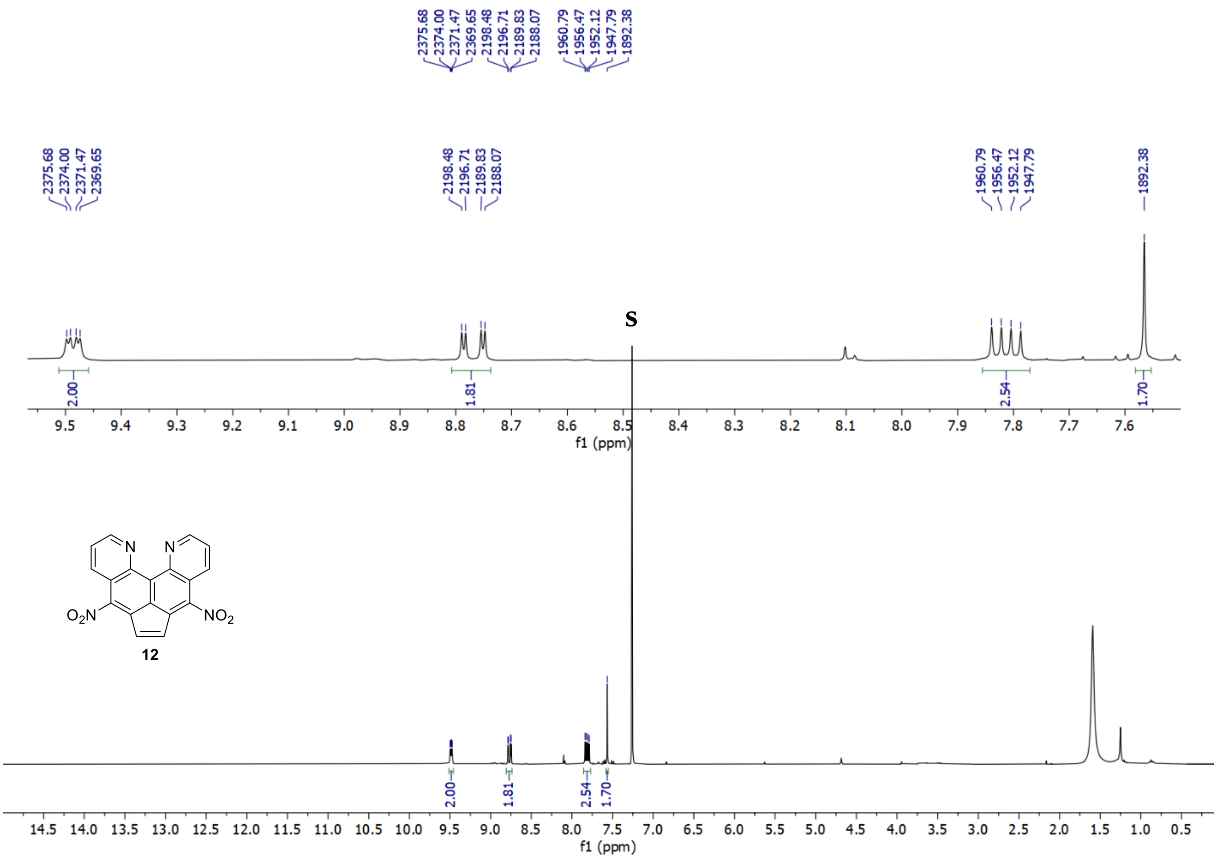

Figure S7:^{1}H NMR spectrum of **12** (250 MHz, $CDCl_3$).

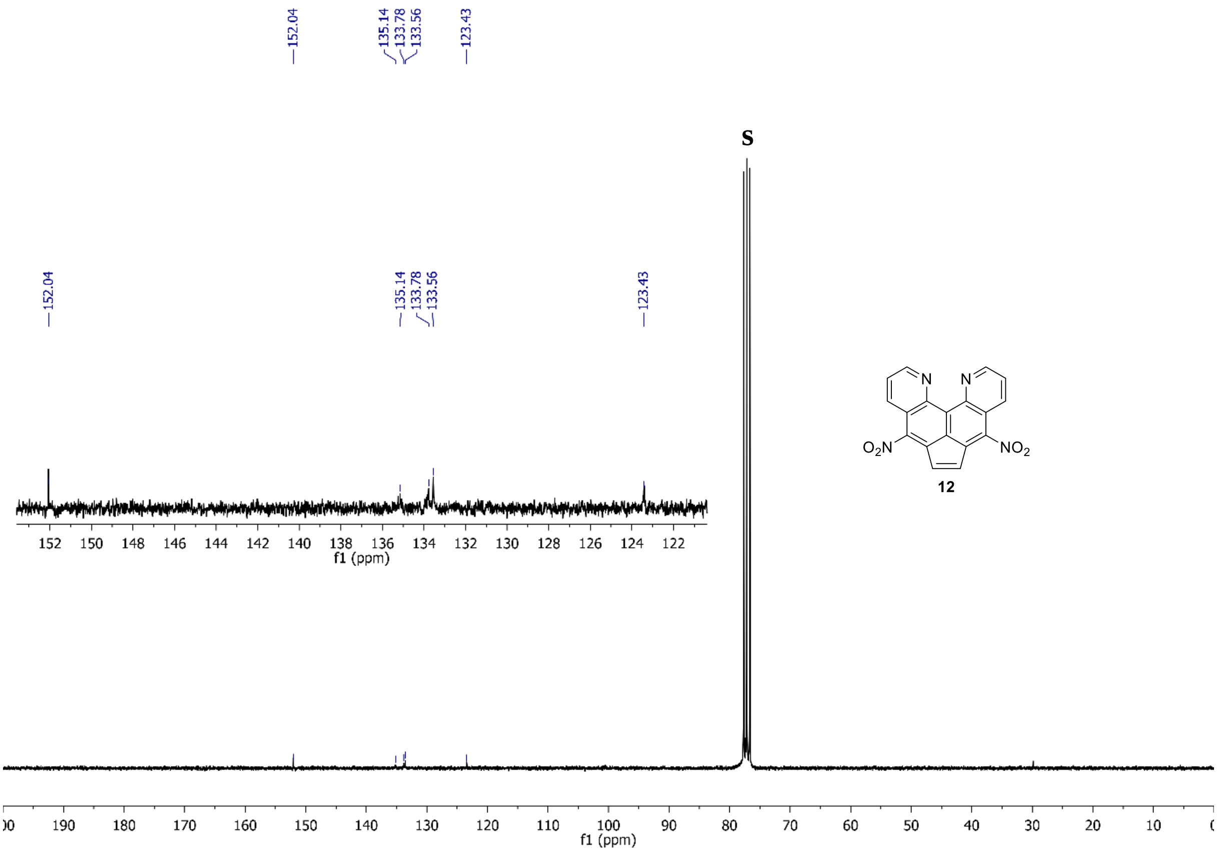

Figure S8:^{13}C{^{1}H} NMR spectrum of **12** (62.9 MHz, $CDCl_3$).

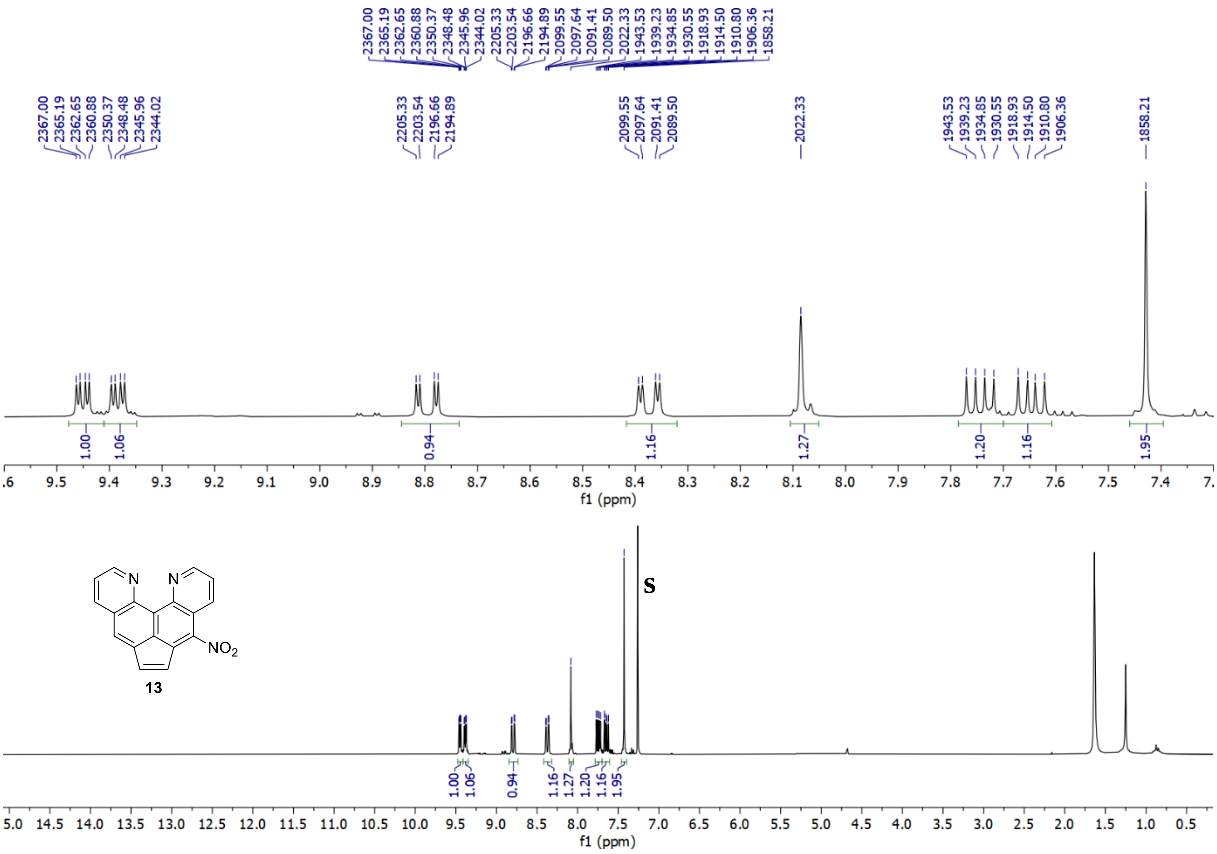

Figure S9: ^{1}H NMR spectrum of **13** (250 MHz, $CDCl_3$).

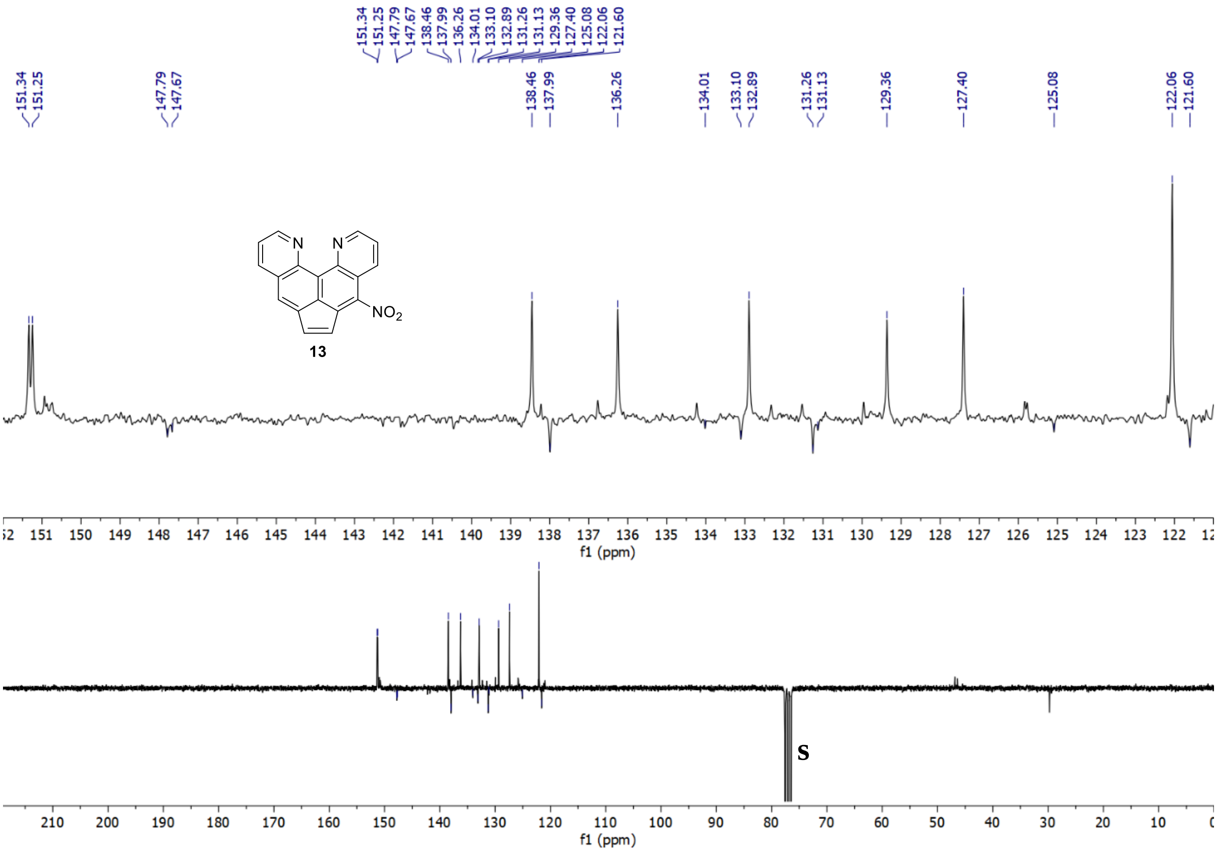

Figure S10: $^{13}C\{^{1}H\}$ APT-NMR spectrum of **13** (62.9 MHz, $CDCl_3$).

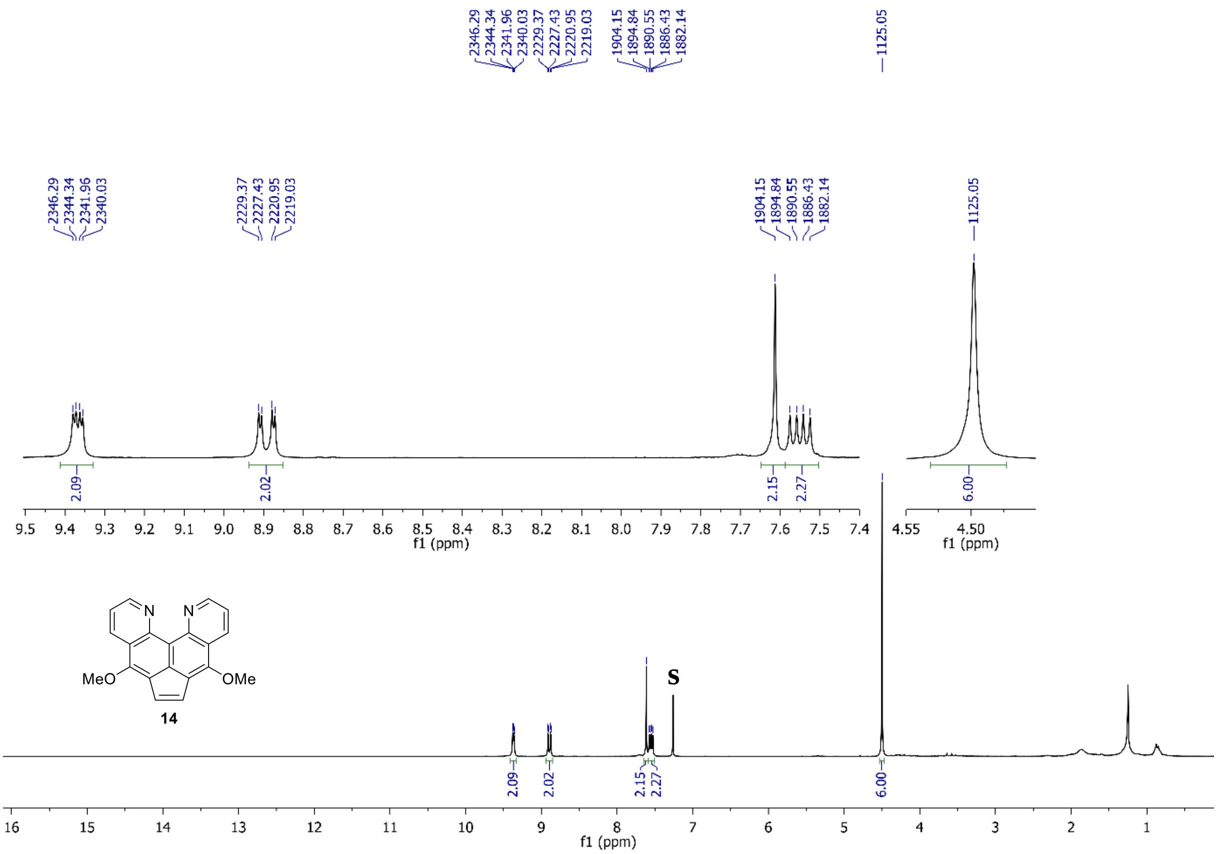

Figure S11: ^{1}H NMR spectrum of **14** (250 MHz, $CDCl_3$).

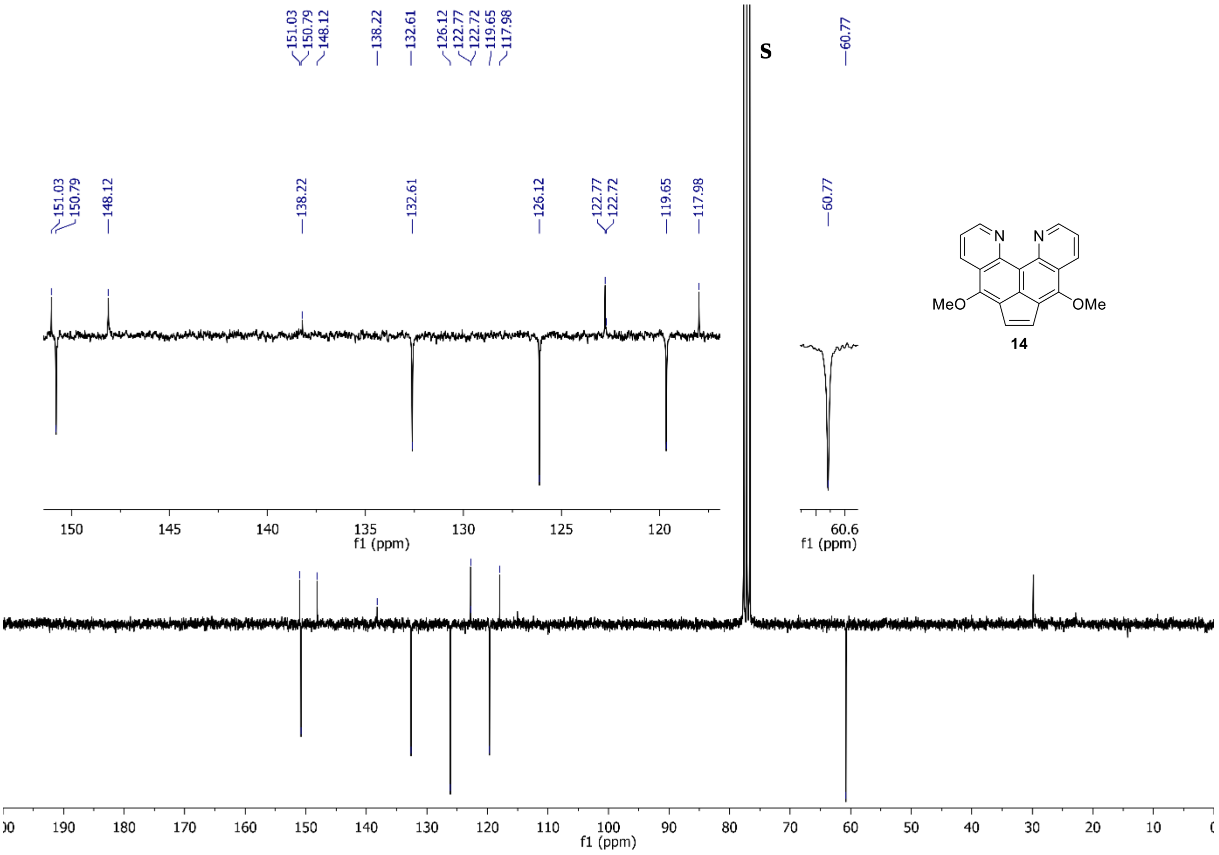

Figure S12: $^{13}C\{^{1}H\}$ APT-NMR spectrum of **14** (62.9 MHz, $CDCl_3$).

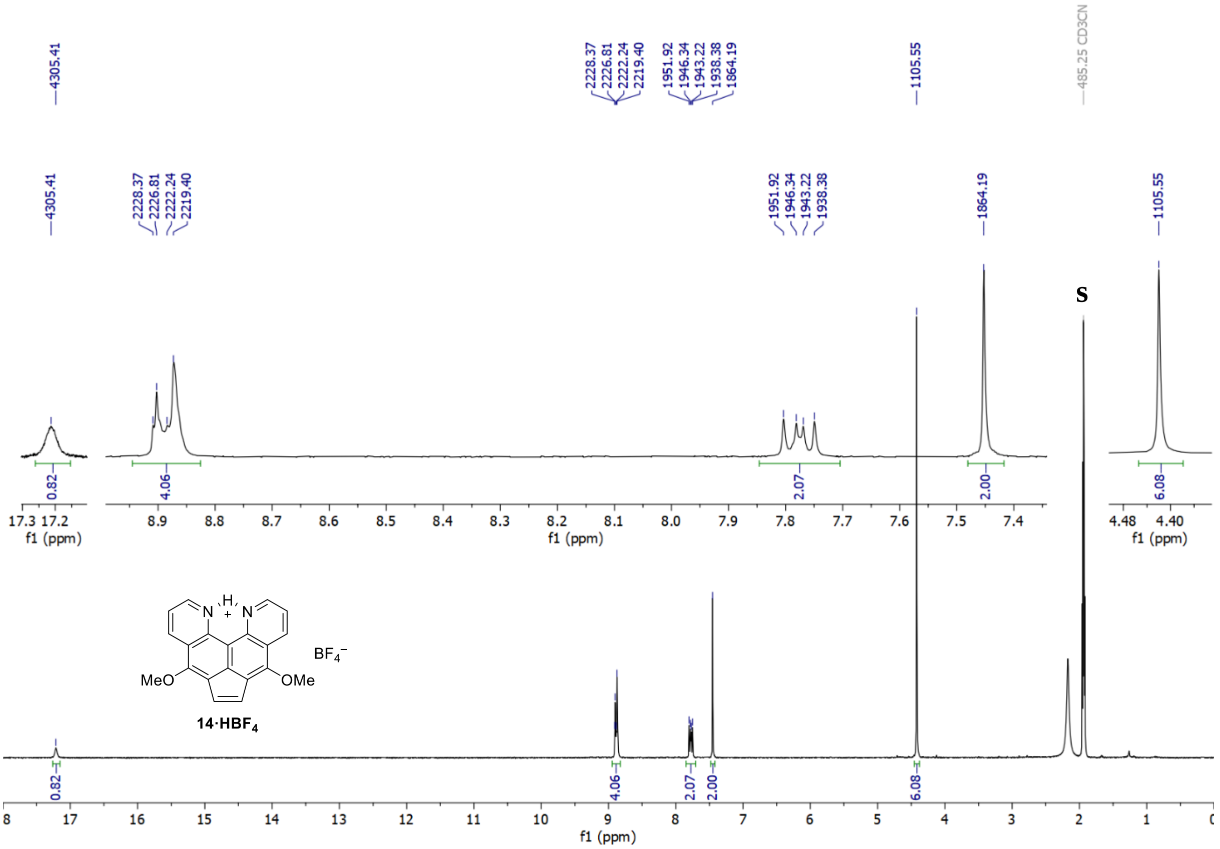

Figure S13: ^{1}H NMR spectrum of **14**·HBF_4 (250 MHz, CD_3CN).

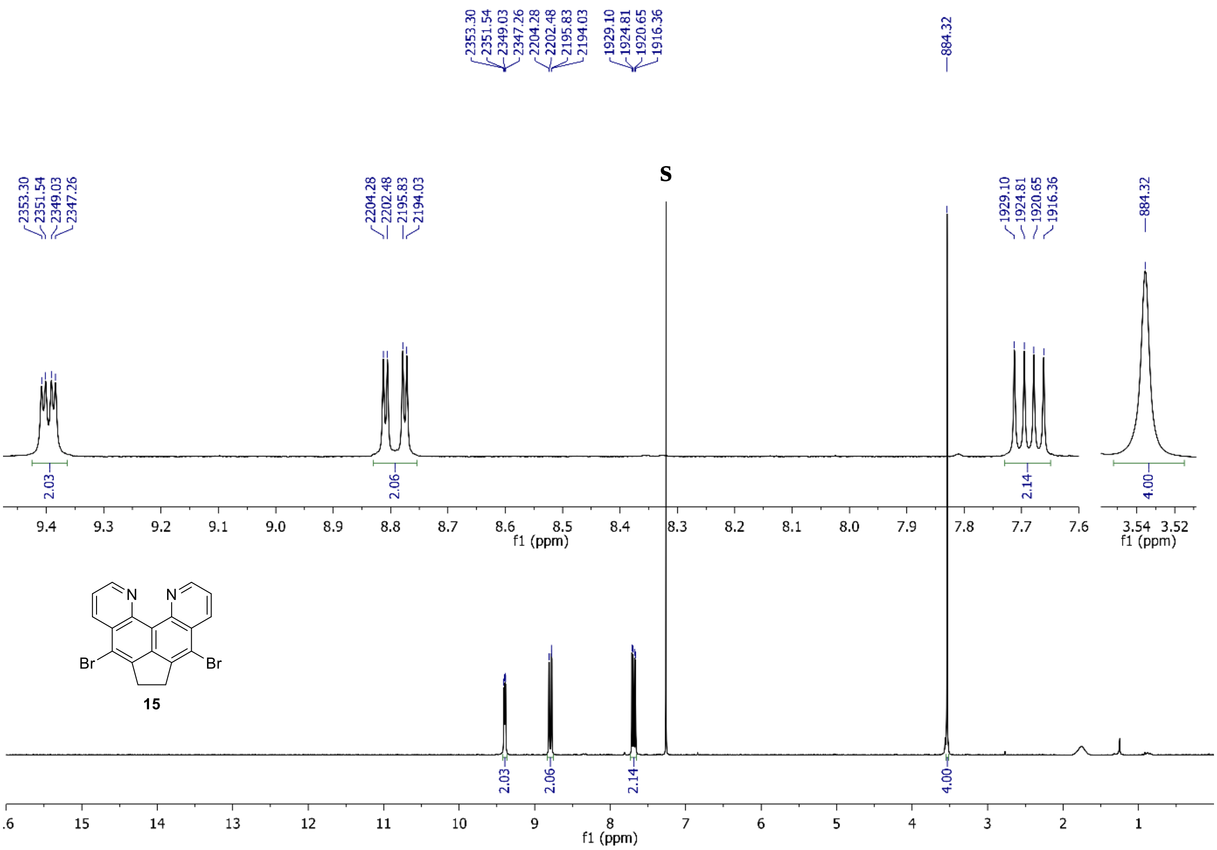

Figure S14: ^{1}H NMR spectrum of **15** (250 MHz, $CDCl_3$).

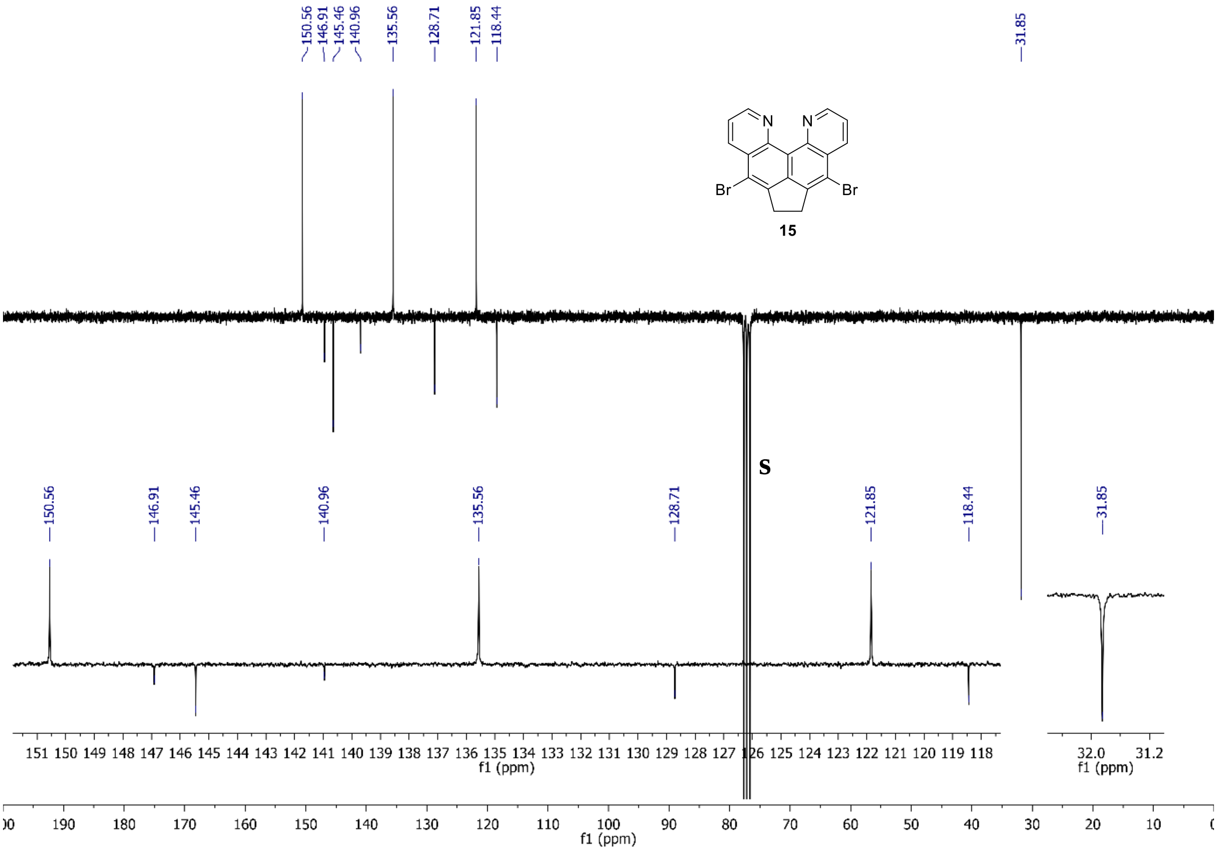

Figure S15: $^{13}C\{^{1}H\}$ APT-NMR spectrum of **15** (62.9 MHz, $CDCl_3$).

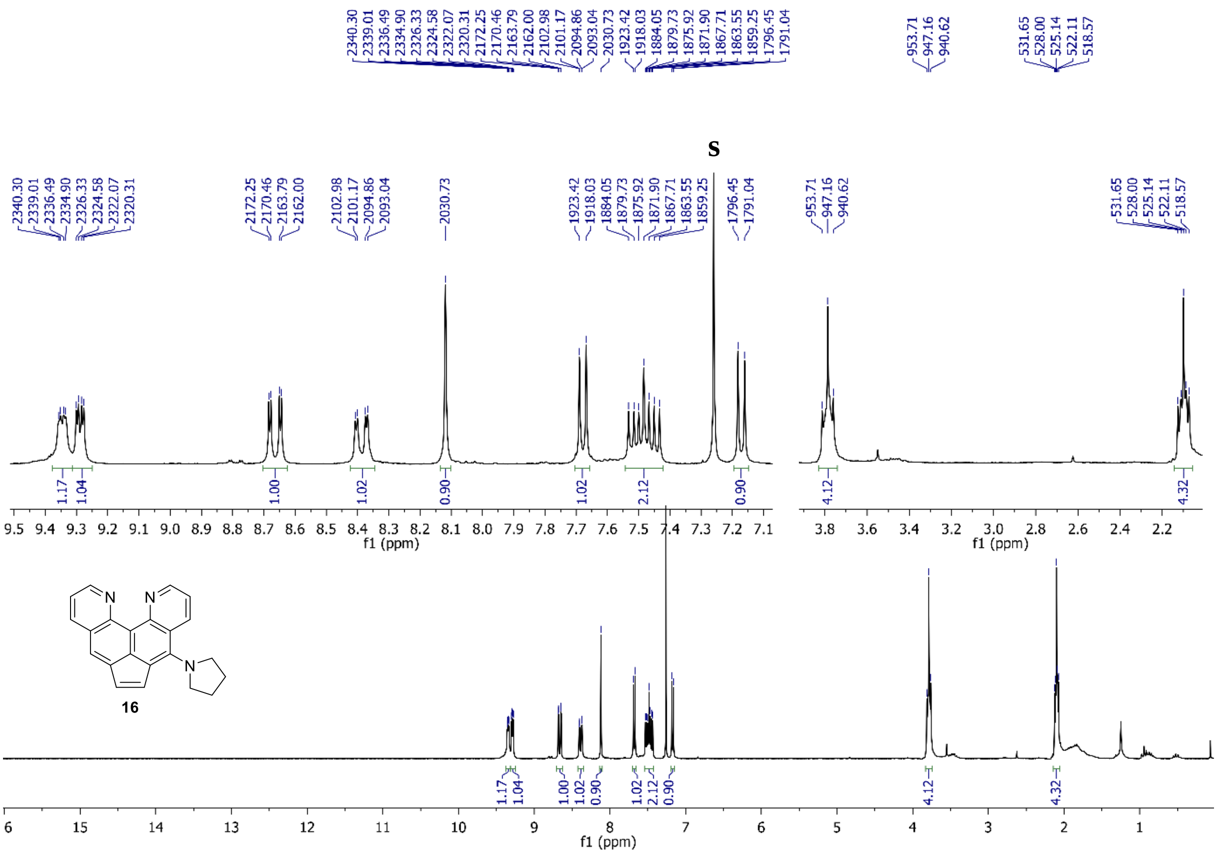

Figure S16: ^{1}H NMR spectrum of **16** (250 MHz, $CDCl_3$).

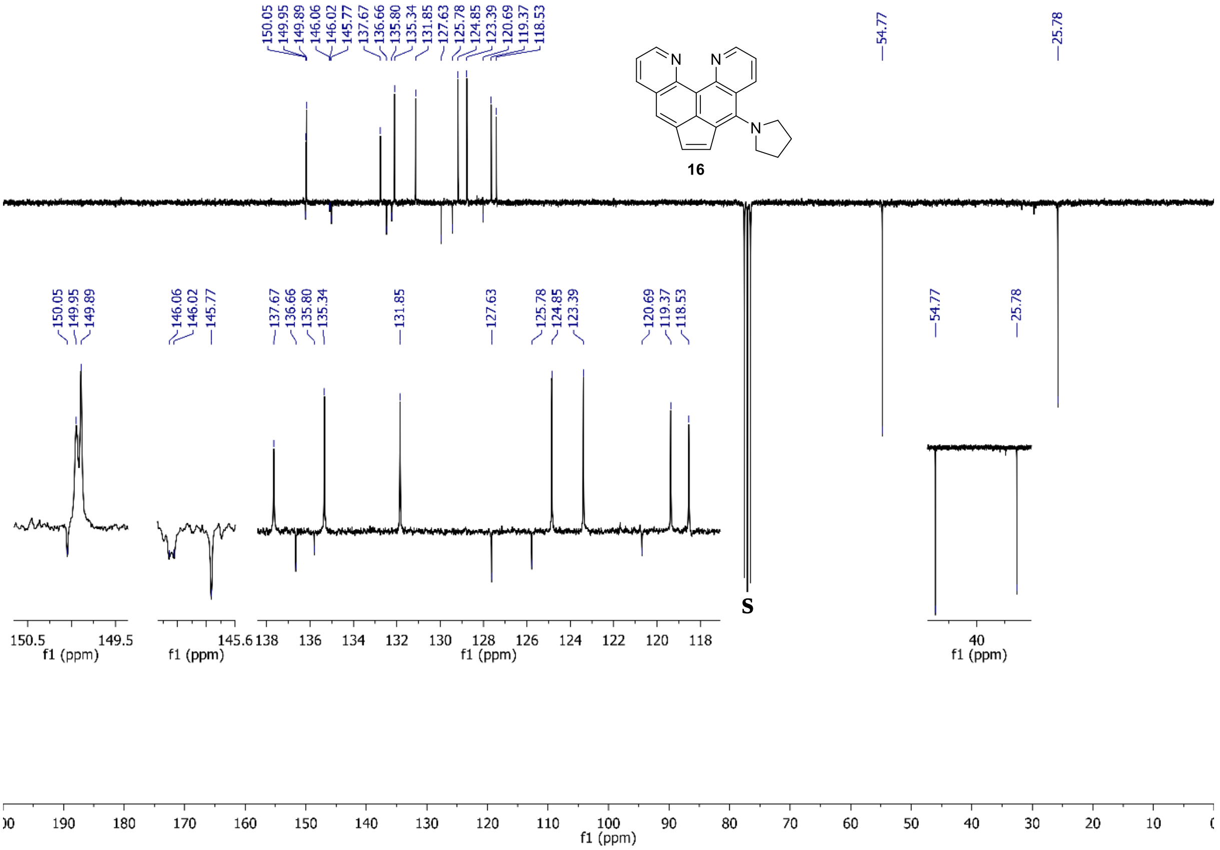

Figure S17: $^{13}C\{^1H\}$ APT-NMR spectrum of **16** (62.9 MHz, $CDCl_3$).

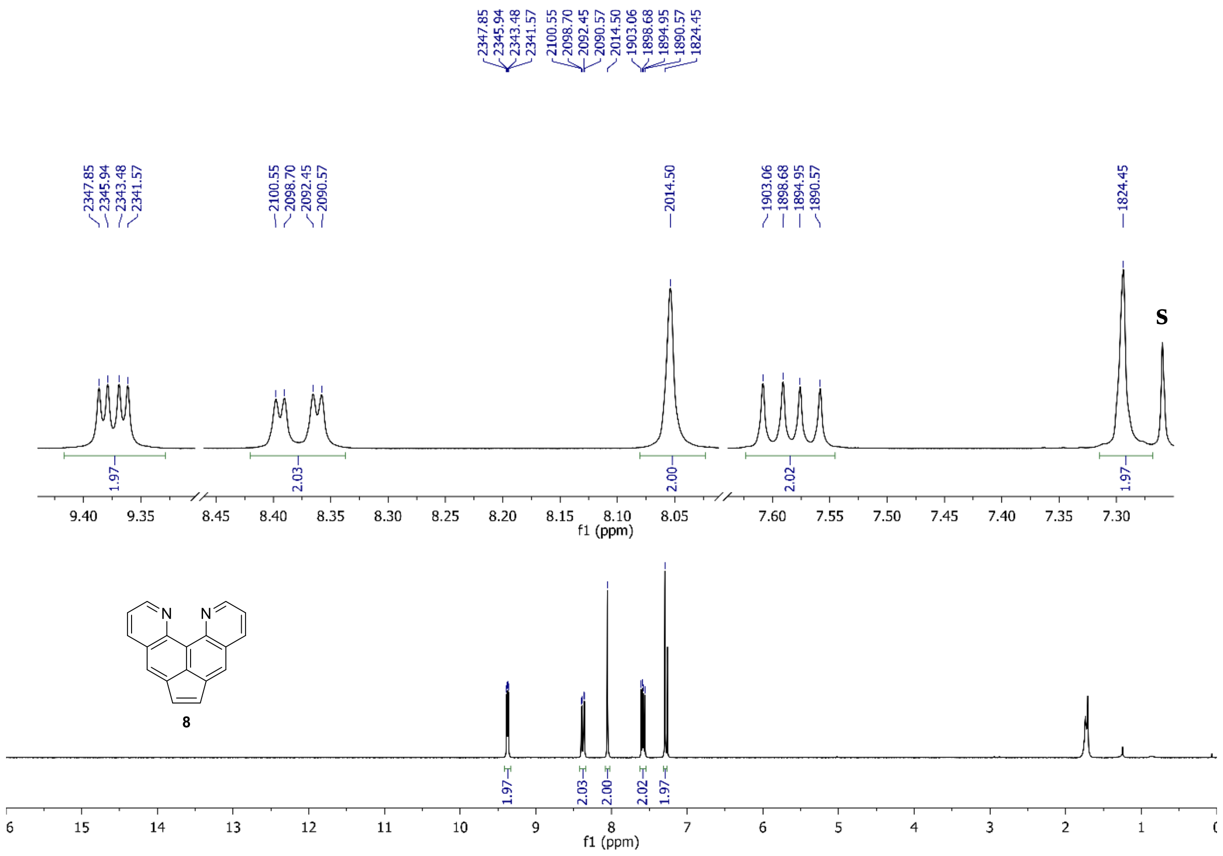

Figure S18: ^{1}H NMR spectrum of **8** (250 MHz, $CDCl_3$).

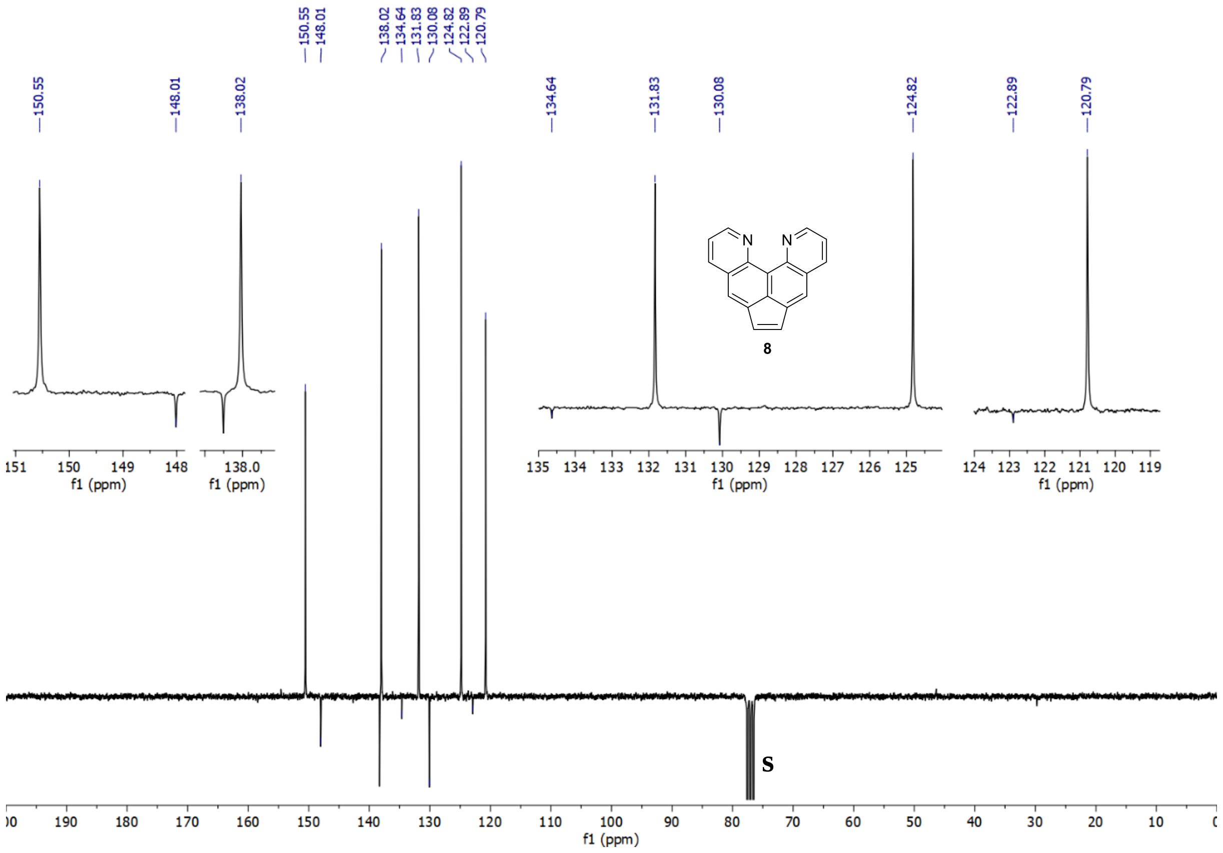

Figure S19: $^{13}C\{^{1}H\}$ APT-NMR spectrum of **8** (62.9 MHz, $CDCl_3$).

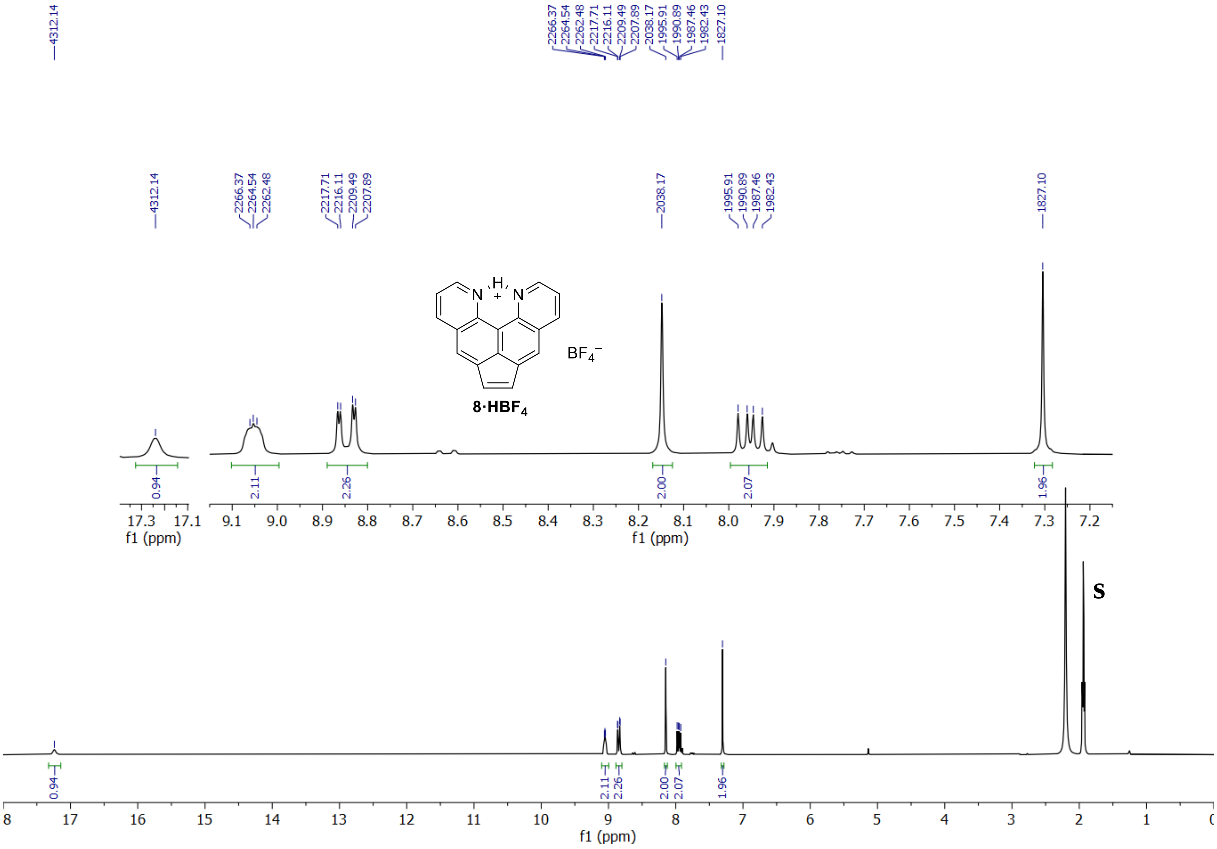

Figure S20: ^{1}H NMR spectrum of **8**·HBF_4 (250 MHz, CD_3CN).

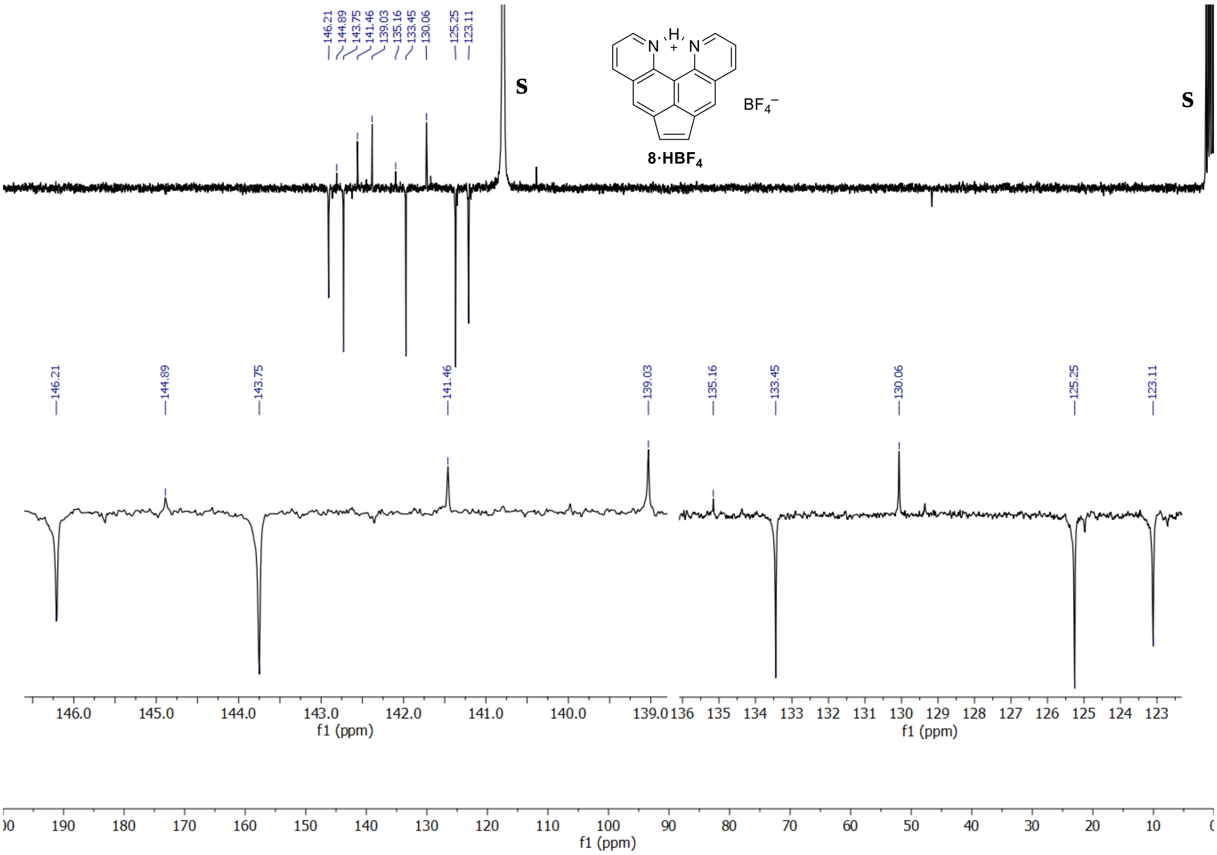

Figure S21: $^{13}C\{^{1}H\}$ APT-NMR spectrum of **8**·HBF_4 (62.9 MHz, CD_3CN).

Crystal data and structure refinement for the studied compounds

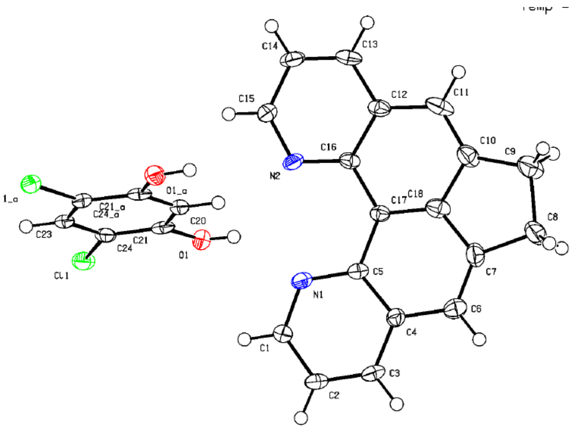<br>**2(5)·(4,6-dichlororesorcinol)**<br>(*P* = 50%, 100 K)	Bond precision: C-C = 0.0071 A<br>Wavelength=1.54184<br>Cell: a=15.1828(18) b=14.5390(17) c=14.0625(16)<br>alpha=90 beta=98.229(12) gamma=90<br>Volume 3072.2(6)<br>Space group I 2/a<br>Moiety formula<br>C18 H12 N2, 0.5(C6 H4 Cl2 O2)<br>Mr 345.79<br>Dx,g cm-3 1.495<br>Z 8<br>Mu (mm-1) 2.288<br>F000 1432.0<br>h,k,lmax 18,17,17<br>Nref 2833<br>Tmin,Tmax 0.891,1.000<br>Data completeness= 0.969 Theta(max)= 69.995<br>R(reflections)= 0.0892(2384)<br>wR2(reflections)= 0.2281(2833)<br>S = 1.079 Npar= 231<br>CCDC 2294253
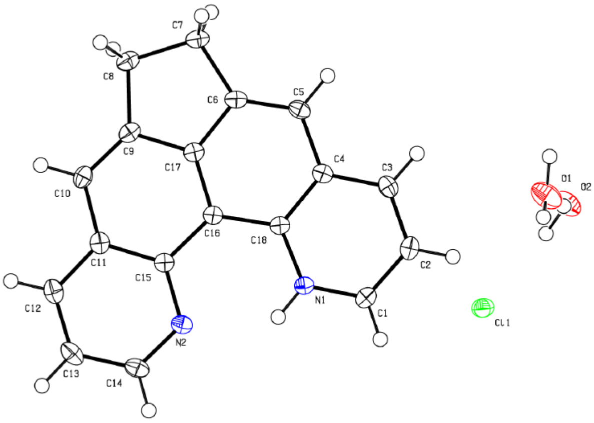<br>**5·HCl·2H₂O** (*P* = 50%, 100 K)	Bond precision: C-C = 0.0017 A<br>Wavelength=1.54184<br>Cell: a=13.8173(2) b=16.4608(2) c=6.8712(1)<br>alpha=90 beta=101.562(1) gamma=90<br>Volume 1531.10(4)<br>Space group P 21/c<br>Moiety formula C18 H13 N2, Cl, 2(H2 O)<br>Mr 328.79<br>Dx,g cm-3 1.426<br>Z 4<br>Mu (mm-1) 2.305<br>F000 688.0<br>h,k,lmax 16,20,8<br>Nref 2905<br>Tmin,Tmax 0.774,1.000<br>Data completeness= 1.000 Theta(max)= 69.985<br>R(reflections)= 0.0282(2826)<br>wR2(reflections)= 0.0804(2905)<br>S = 1.063 Npar= 228<br>CCDC 2294257
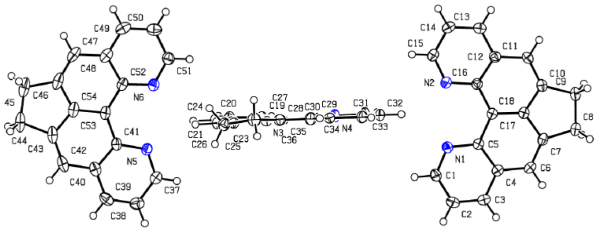<br>**5** (*P* = 50%, 100 K)	Bond precision: C-C = 0.0016 A<br>Wavelength=1.54184<br>Cell: a=11.1435(1) b=12.8407(1) c=13.8368(1)<br>alpha=74.524(1) beta=83.643(1) gamma=77.720(1)<br>Volume 1861.40(3)<br>Space group P -1<br>Moiety formula C18 H12 N2<br>Mr 256.30<br>Dx,g cm-3 1.372<br>Z 6<br>Mu (mm-1) 0.636<br>F000 804.0<br>h,k,lmax 13,15,16<br>Nref 7057<br>Tmin,Tmax 0.901,1.000<br>Data completeness= 0.999 Theta(max)= 69.984<br>R(reflections)= 0.0350(6451)<br>wR2(reflections)= 0.0968(7057)<br>S = 1.022 Npar= 541<br>CCDC 2294256

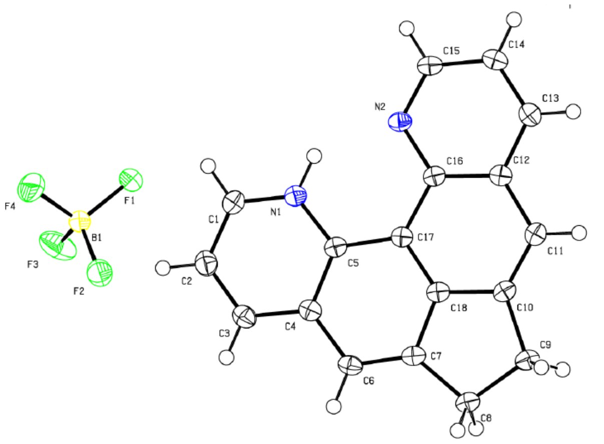<br>**5·HBF$_4$** (P = 50%, 100 K)	Bond precision: C-C = 0.0020 A<br>Wavelength=1.54184<br>Cell: a=11.4104(2) b=6.7594(1) c=18.8779(4)<br>alpha=90 beta=95.323(2) gamma=90<br>Volume 1449.73(5)<br>Space group P 21/n<br>Moiety formula C18 H13 N2, B F4<br>Mr 344.11<br>Dx,g cm-3 1.577<br>Z 4<br>Mu (mm-1) 1.105<br>F000 704.0<br>h,k,lmax 14,8,23<br>Nref 3065<br>Tmin,Tmax 0.735,1.000<br>Data completeness= 0.990 Theta(max)= 77.455<br>R(reflections)= 0.0438(2731)<br>wR2(reflections)= 0.1257(3065)<br>S = 1.030 Npar= 234<br>CCDC 2294254
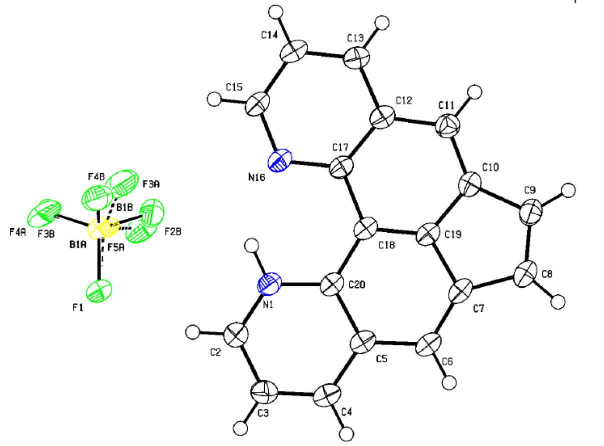<br>**8·HBF$_4$** (P = 50%, 100 K)	Bond precision: C-C = 0.0028 A<br>Wavelength=1.54184<br>Cell: a=6.7710(5) b=10.3820(7) c=11.4650(6)<br>alpha=116.396(6) beta=90.535(6) gamma=94.680(6)<br>Volume 718.58(9)<br>Space group P -1<br>Moiety formula C18 H11 N2, B F4<br>Mr 342.10<br>Dx,g cm-3 1.581<br>Z 2<br>Mu (mm-1) 1.115<br>F000 348.0<br>h,k,lmax 8,13,14<br>Nref 3001<br>Tmin,Tmax 0.564,1.000<br>Data completeness= 0.986 Theta(max)= 76.762<br>R(reflections)= 0.0550(2583)<br>wR2(reflections)= 0.1643(3001)<br>S = 1.052 Npar= 267<br>CCDC 2294255

Crystal packing in salts 5·HBF$_4$ and 8·HBF$_4$

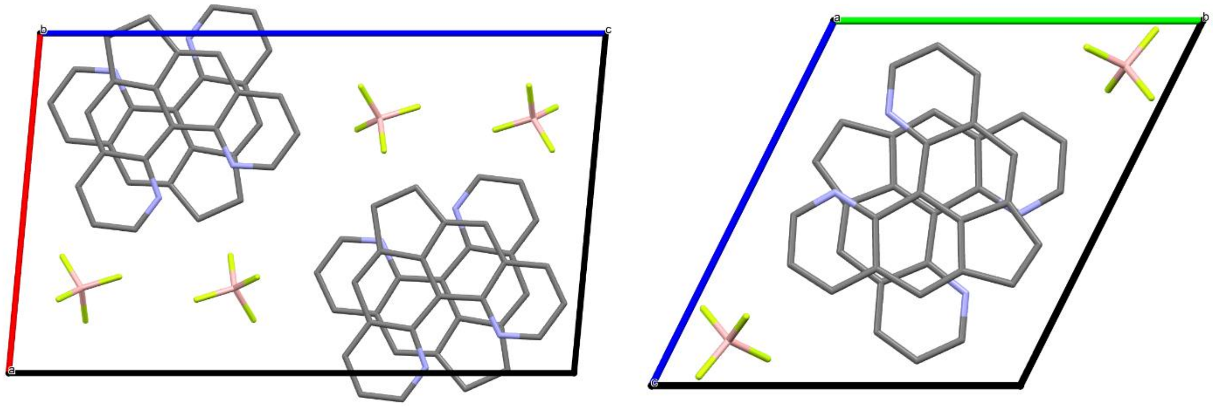

Figure S22 Crystal packing motifs in salts **5**·HBF$_4$ (*left*) and **8**·HBF$_4$ (*right*) showing the formation of dense columns consisting of stacking flat heterocyclic cations (along the *b*-axis for **5**H$^+$ and along the *a*-axis for **8**H$^+$). Hydrogen atoms are omitted for clarity. The shortest distance between the antiparallel π-systems of two molecular planes is 3.372 Å for **5**·HBF$_4$ and 3.328 Å for **8**·HBF$_4$).
